# Transcriptomic analysis reveals candidate genes associated with salinity stress tolerance during the early vegetative stage in fababean genotype, Hassawi-2

**DOI:** 10.1038/s41598-023-48118-0

**Published:** 2023-12-01

**Authors:** Muhammad Afzal, Salem S. Alghamdi, Muhammad Altaf Khan, Sulieman A. Al-Faifi, Muhammad Habib ur Rahman

**Affiliations:** 1 Department of Plant Production, College of Food and Agricultural Science, King Saud University, 11451 Riyadh, Saudi Arabia; 2 INRES Institute of Crop Science and Resources Conservation INRES University of Bonn, Bonn, Germany; 3 Seed Science and Technology, Institute of Plant Breeding and Biotechnology, MNS University of Agriculture, Multan, Pakistan

**Keywords:** Biotechnology, Molecular biology, Plant sciences

## Abstract

Abiotic stresses are a significant constraint to plant production globally. Identifying stress-related genes can aid in the development of stress-tolerant elite genotypes and facilitate trait and crop manipulation. The primary aim of this study was to conduct whole transcriptome analyses of the salt-tolerant faba bean genotype, Hassawi-2, under different durations of salt stress (6 h, 12 h, 24 h, 48 h, and 72 h) at the early vegetative stage, to better understand the molecular basis of salt tolerance. After de novo assembly, a total of 140,308 unigenes were obtained. The up-regulated differentially expressed genes (DEGs) were 2380, 2863, 3057, 3484, and 4820 at 6 h, 12 h, 24 h, 48 h, and 72 h of salt stress, respectively. Meanwhile, 1974, 3436, 2371, 3502, and 5958 genes were downregulated at 6 h, 12 h, 24 h, 48 h, and 72 h of salt stress, respectively. These DEGs encoded various regulatory and functional proteins, including kinases, plant hormone proteins, transcriptional factors (TFs) basic helix-loop-helix (*bHLH), Myeloblastosis (MYB),* and *(WRKY*), *heat shock proteins* (*HSPs*), *late embryogenesis abundant* (*LEA*) proteins, dehydrin, antioxidant enzymes, and aquaporin proteins. This suggests that the faba bean genome possesses an abundance of salinity resistance genes, which trigger different adaptive mechanisms under salt stress. Some selected DEGs validated the RNA sequencing results, thus confirming similar gene expression levels. This study represents the first transcriptome analysis of faba bean leaves subjected to salinity stress offering valuable insights into the mechanisms governing salt tolerance in faba bean during the vegetative stage. This comprehensive investigation enhances our understanding of precise gene regulatory mechanisms and holds promise for the development of novel salt-tolerant faba bean salt-tolerant cultivars.

## Introduction

Salt stress is a significant factor that limits crop production worldwide, affecting over 800 million hectares of land globally, with this number rising due to poor agricultural practices^1^. Salinity profoundly impacts various plant growth attributes, development, yield, grain quality, and nutrient composition^2^. In addition to osmotic and ion imbalance stress, salt stress also causes secondary stresses, such as nutritional imbalance and oxidative stress in glycophytes^3^. Salt stress can alter gene expression to indirectly affect cell wall properties, while physical interactions of Na^+^ with cell wall components can modify their chemical characteristics^4^. Ultimately, salt stress affects the control of metabolism, gene expression, and the function of proteins in plants. Additionally, salinity stress affects cell wall composition, transport mechanisms, cell size and shape, and root architecture^5^. Faba bean (*Vicia faba* L.) is the third most important cool-season food legume crop cultivated in over 60 countries worldwide^6^. It is an ancient crop that has been cultivated in the Mediterranean region for a long time^7^. Global grain legume crop with 490Tg produced over 250 million hectares (Mha) (FAOSTAT 2021). It is a diploid species (2n = 12) with commercial importance, but there has been modest progress in developing a thorough genetic understanding of the crop. Faba bean has the largest genome (13.4 Gb) among grain legumes^8^. Faba bean genome has been recently published^9^ which will likely open new avenues to plant breeders and geneticists. Faba bean is an essential feed for cattle and a source of proteins for humans due to its high nutritional content^10^. In semi-arid regions, salinity severely affects the soil and limits faba bean production^11^. Faba bean is a salt-sensitive crop, and its yield can decrease up to 50% under high salinity levels^12^. Furthermore, salinity has a hazardous effect on plant growth, rhizobium symbiosis, the formation of root nodules, and legumes' ability to fix nitrogen^13^. However, the faba bean draft genome has recently been assembled^9^ and a detailed transcript profile of the faba bean species at the early vegetative stage could be necessary to reveal candidate genes and fundamental groups involved in the regulation of the crop's most crucial agronomic characteristics. It could also offer a practical base from which to assemble a species-specific gene map. In Saudi Arabia, faba beans are increasingly becoming part of the human diet, with both fresh and dried kernels being consumed. To meet the growing demand, the Kingdom of Saudi Arabia (KSA) imports about 100 million tons of seed legumes annually, costing around 145 million SAR (Saudi Arabia Ministry of Economy and Planning, 2015). As the market for faba beans expands in KSA, farmers are becoming more interested in selecting genes with agricultural significance, such as growth qualities, the absence of tannins, and stress tolerance. However, the yield of faba bean is impacted by various abiotic factors, such as drought^14^, and salinity^15^.

A molecular understanding of the stress mechanism is essential for breeding stress-related germplasm. Therefore, the plant growth cycle based on vegetative growth is critical in ensuring plant survival and reproduction. Hence, salt-tolerant cultivars of faba bean are important for selective breeding to enhance production^16^. RNA sequencing is an in-depth method for profiling the transcriptome and examining certain biological processes under specific conditions^17^. The application of RNA-seq provides information about gene characterization, functional genomic studies, and gene expression analysis, which is widely reported in different crop plants, including chickpea^18^, rice^19^, field pea^20^ and pigeon pea^21^. However, in faba bean, genome-wide association studies (GWAS) were reported in leaves, stems, and seed stages with different gene expression levels^22,23^. RNA-Seq is a highly reproducible, highly accurate, and dynamically flexible quantification technique used to determine drug response, biomarker identification, basic medical research, and drug research and development (R&D)^24^. In addition, RNA-Seq was also used for gene expression analysis and differential gene expression analysis pattern and gene ontology (GO) classification^25^. The current study was designed for transcriptome profiling of the salt-tolerant faba bean cultivar Hassawi-2 under five different salt stress exposures (6 h, 12 h, 24 h, 48 h, and 72 h) at early vegetative stage in leaf using next-generation RNA-sequencing techniques.

## Results

### RNA sequencing and data evaluation

cDNA libraries were generated from leaves of the salt tolerant Faba bean genotype, Hassawi-2, at the early vegetative stage, and treated with 200 mM NaCl salt stress for control and five salt stress exposure times (6h, 12h, 24h, 48h, 72h) to investigate changes in gene expression. Each sample consist of three biological replicates. Library construction and sequencing were performed using Illumina HiSeq sequencing technology for a total of seventeen libraries. The statistics for sequencing data (raw and clean reads) for each library at each stress exposure time, are presented (Supplementary Table S1). In total, 940 million clean Illumina paired-end RNA-Seq reads were obtained, with Q20 and Q30 values exceeding 98.40 and 95.27 respectively, of all raw data. Only clean reads from each library were used for subsequent analysis. Each library was individually analyzed and then combined for analysis, resulting in a total of 140,308 unigenes and 174,051 transcripts obtained after de novo assembly with an average contig length of 674.82 bp and 640.19 bp, respectively. The de novo assembly statistics are provided in Table 1.

**Table 1:** Statistics of assembled transcriptome data.

Assembly	number	GC (%)	N50	Avg. contig length (bp)	Total assembled bases (bp)
Transcripts	174,051	38.31	941	640.19	111,425,427
Unigenes	140,308	37.78	1038	674.82	94,683,004

**Figure 1:**
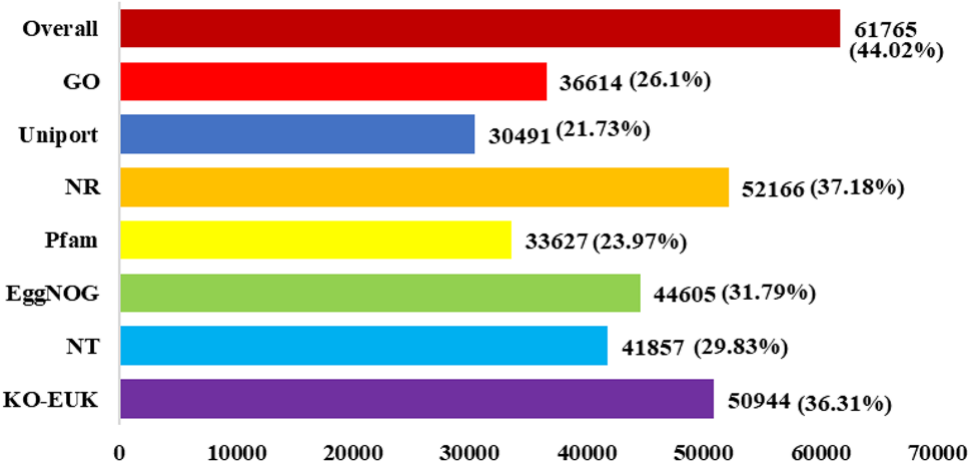
Functional annotations result of All-unigenes from various databases.

The unigenes obtained after assembly were functionally annotated using BLASTX against NCBI non-redundant Protein (NR), Swiss-Prot, Kyoto Encyclopedia of Genes and Genomes (KEGG), Gene Ontology (GO), and (Eukaryotic Orthologous Groups) COG/KOG (E-value < 1e−5). Overall, an annotation ratio of 44.02% was achieved, accounting for 61,765 of the totals 140,308 unigenes were annotated in at least one public database (Fig. 1). Specifically, 36,614 (26.1%) were linked to GO terms, 30,491 (21.73%) were matched with UniProt, 52,166 (37.18%) had hits in the NR database, 33,627 (23.97%) were associated with protein families (Pfam), and 44,605 (31.79%) were categories in Evolutionary Genealogy of Genes: Non-supervised Orthologous Groups (EggNOG). The NCBI Nucleotide (NT) database contains records for 41,857 (29.83%) unigenes, while 50,944 (36.31%) were found through KO_EUK (Fig. 1). The detailed functional annotation results from all databases are presented in Supplementary Table (S1).

**Figure 2:**
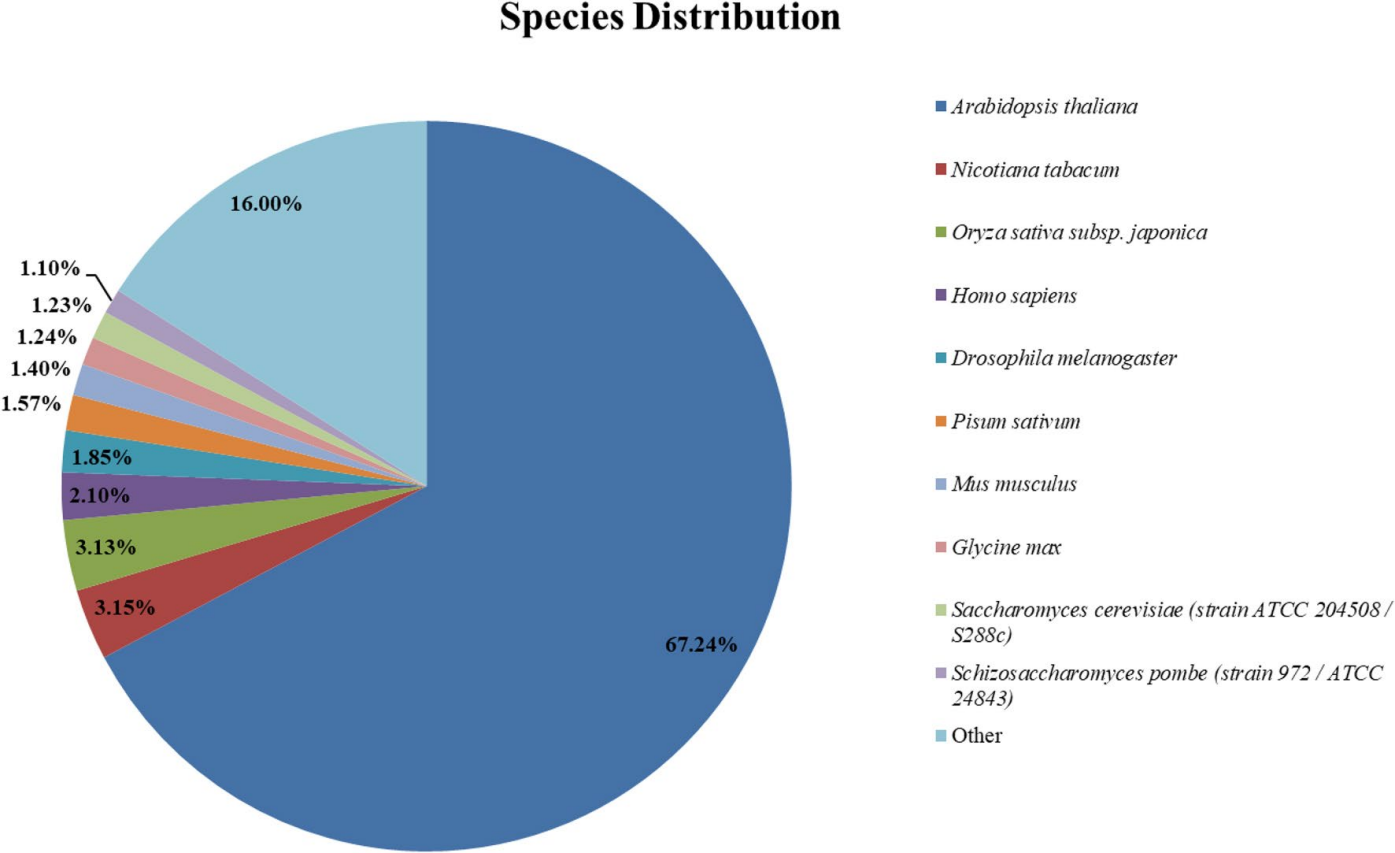
Top-hit species distribution similarities using Basic local alignment search tool (BLAST).

The top eight species distribution for functional annotations are *Arabidopsis thaliana* (67.24%), *Nicotiana tabacum* (3.15%), *Oryza sativa subsp. Japonica* (3.13%), *Homo sapiens* (2.10%), *Drosophila melanogaster* (1.85%), *Pisum sativum* (1.57%), *Mus musculus* (1.40%), *Glycine max* (1.23%), and others include (16%) (Fig. 2).

### Differential gene expression (DEGs) at different salt stress exposure

**Figure 3:**
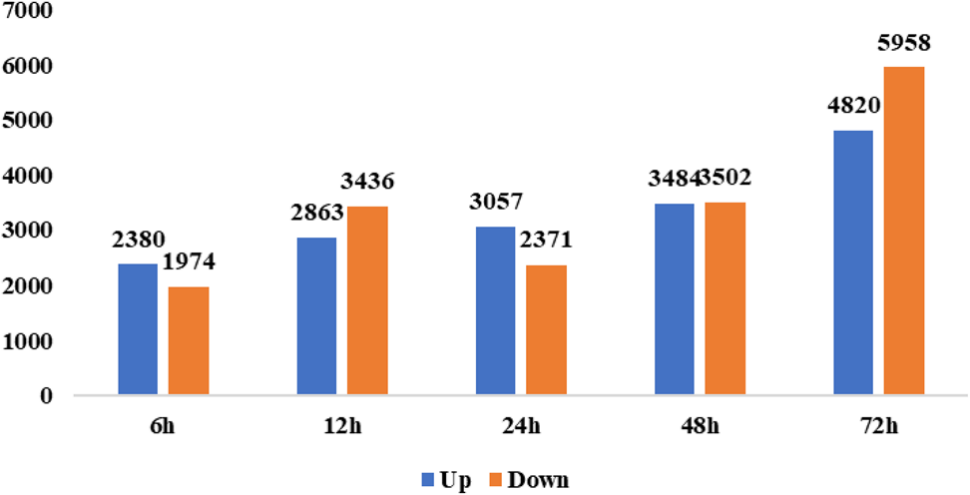
Number differentially expressed genes (up- and down-regulated) in Faba bean leaves at five time points (6 h, 12 h, 24 h, 48 h, and 72 h) of 200 mM salt stress.

The clean reads obtained from the 17 libraries were mapped to the assembled unigenes, resulting in mapping ratios ranging from 76.23% to 82.22% (Table S1). Gene expression levels are presented in Table S3; Table S4; Table S5; Table S6; Table S7. To elucidate both the commonalities and distinctions between the control and salt stress-exposed samples, and to conduct a deeper analysis of their biological functions, an expression analysis was performed across various time points of salt stress exposure (6 h, 12 h, 24 h, 48 h, and 72 h). The primary objective was the identification of differentially expressed genes (DEGs) in response to salt stress within leaf samples. To accomplish this, transcripts with exceptionally low expression levels were excluded after an initial analysis of the unigene expression levels. The differentially expression of genes (DEGs) at each of the five stress exposure time points for leaf samples is depicted in Fig. 3. Using a fold change threshold of (|FC|> = 2), we observed 2380, 2863, 3057, 3484, and 4820 upregulated DEGs and 1974, 3436, 2371, 3502, and 5958 downregulated DEGs at 6 h, 12 h, 24 h, 48 h, and 72 h, respectively (Fig. 3).

The number of DEGs genes for each contrasting pair (6 h/12 h, 12 h/24 h, 24 h/48 h, 48 h/72 h) was determined (Fig. S1). To investigate whether delayed salinity stress impacts gene expression in a plant's ability to tolerate salinity during the vegetative stage, pairwise comparisons were conducted among 6 h/12 h, 12 h/24 h, 24 h/48 h, and 48 h/72 h. Interestingly, differences were observed at each delayed time under salt stress conditions. At 12 h, 2823 unique DEGs genes were identified compared to 766 DEGs at 6 h, while 1369 genes were common to both times points. Similarly, the most abundant genes, 2417 in total, was present at 12 h compared to 1103 genes at 24 h, with 1775 genes shared between 12 h/24 h. The majority of common genes 1828 (42%) were found between 24 h/48 h, whereas 1050 DEGs were found at 24 h salt stress exposure relative to 48 h (1476). At 72 h, the maximum number of unique DEGs genes (6206) was found, while 715 unique DEGs were observed at 48 h, and 2589 overlapped genes were identified 48 h/72 h (Fig. S1). Based on the results, the significant variation in gene expression numbers may be attributed to the distinct mechanisms of salinity tolerance adopted at different salt stress delayed salt stress time points. The findings indicate that numerous genes were temporarily upregulated at the leaf stage under delayed salt stress conditions relative to the control (Fig. S2).

### Analysis of differentially expressed genes (DEGs)

#### Annotation on gene ontology (GO) database

GO annotations were employed to determine the functions of DEGs in different comparisons. Each salt stress exposure time (6 h, 12 h, 24 h, 48 h, 72 h) was categorized into molecular function, biological processes, cellular components, and no-hit data based on GO terms (Fig. 3). The distribution of GO term blast results across biological processes was 23.86%, 26.31%, 25.86%, 26.07%, and 25.12%; for cellular components was 26.16%, 29.54%, 28.36%, 28.03%, and 28.28%; molecular functions were 25.2%, 27.83%, 26.04%, 26.44%, and 25.97%; while no-hit data represented 24.78%, 16.32%, 19.74%, 19.46%, and 20.63% at 6 h, 12 h, 24 h, 48 h, and 72 h of salt stress exposure (200 mM), respectively (Fig. 3).

**Figure 4:**
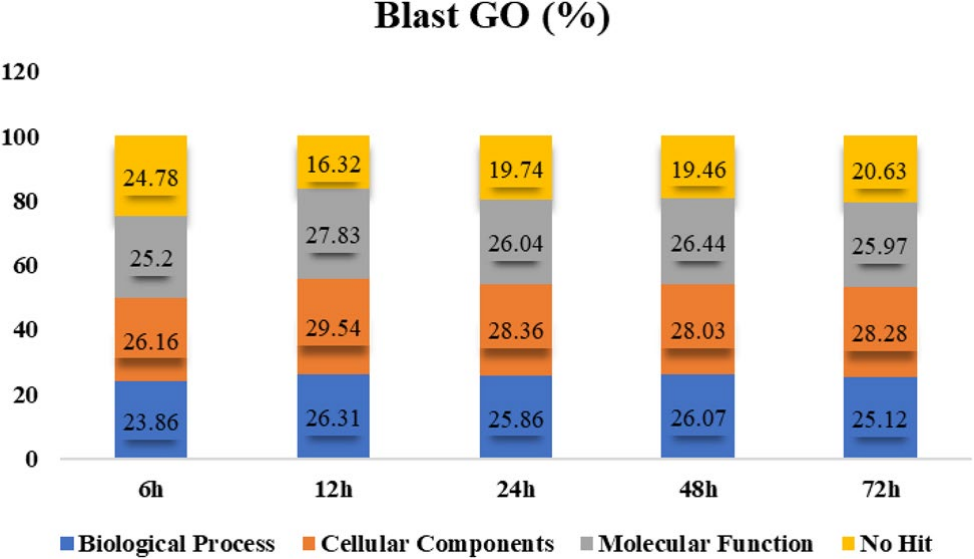
Gene ontology functional distribution categories of differentially expressed genes (%) of biological processes, cellular components, molecular function, and no-hit data assigned at different time points (6 h, 12 h, 24 h, 48 h and 72 h) of 200 mM salt stress.

**Figure 5:**
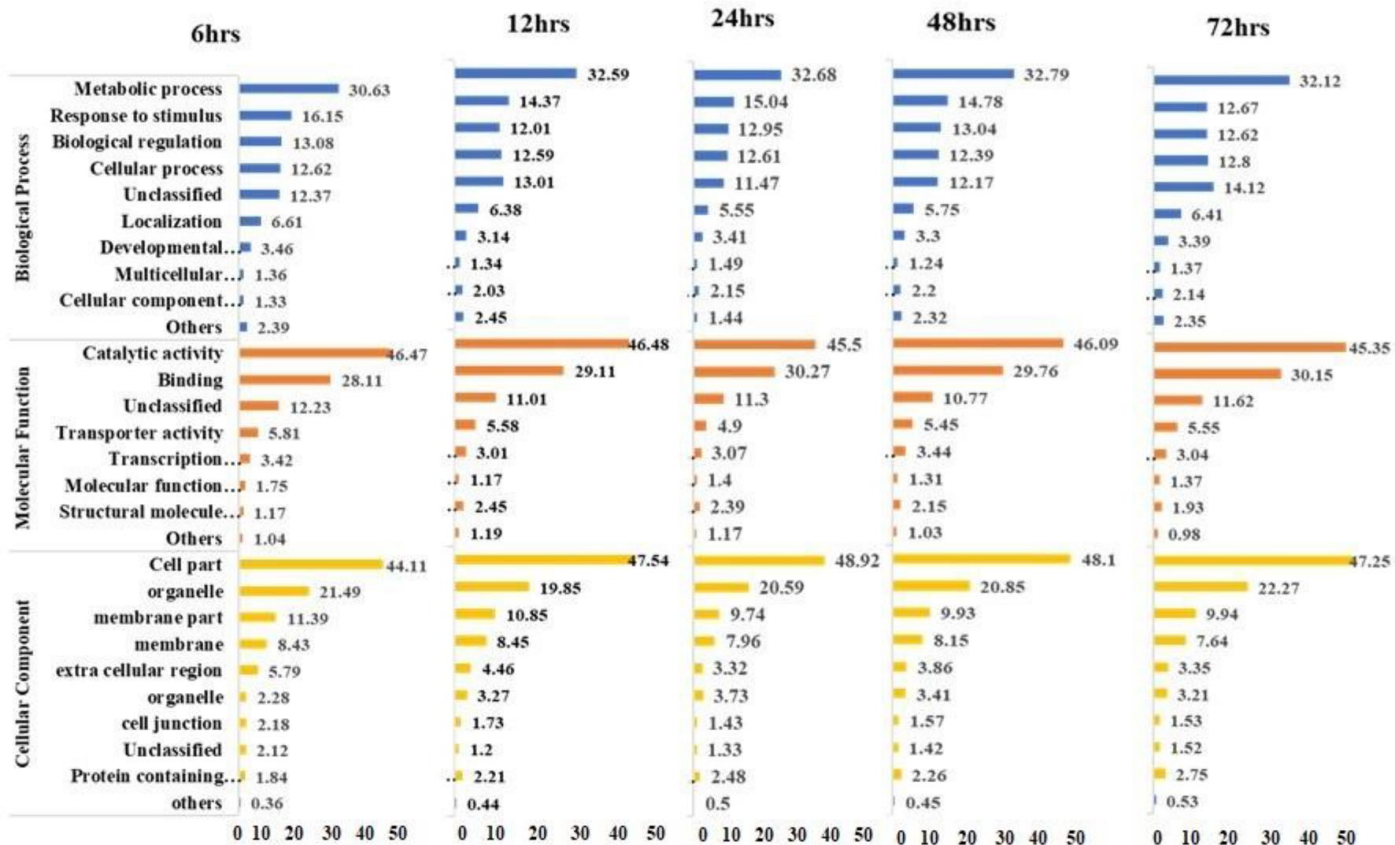
Distribution of gene ontology terms into biological process, molecular function, and cellular components at 6 h, 12 h, 24 h, 48 h and 72 h time points of 200 mM salt stress. X-axes indicate the number of DEGs (%) and Y-axis indicate the Gene ontology terms.

The individual GO terms for biological processes, molecular function and cellular components were also documented (Fig. 4). The GO terms related to metabolic processes, biological regulation, response to stimuli, and cellular processes were the most prevalent among the unigenes at each salinity stress level. In terms of molecular functions, the most abundant terms included catalytic activity, binding, transporter activity, and transcription regulator activity across at all five stress levels. Similarly, the most common cellular components described by GO terms were cell part, organelle, membrane part, and membrane for all stress levels. A detailed comparison of the GO term profiles revealed a strong resemblance among them, indicating a high degree of consistency, but with different numbers of DEGs genes (Fig. 5).

### KEGG enrichment analysis

**Figure 6:**
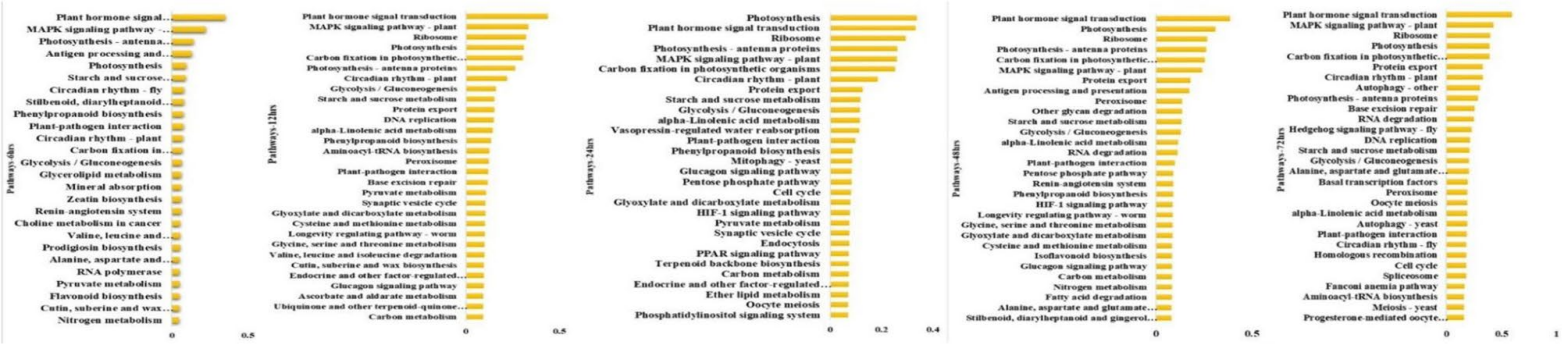
Top 30 KEGG pathways classification of differentially expressed genes (DEGs) at different time points (6 h, 12 h,24 h, 48 h, 72 h) of 200 mM salt stress.

The Kyoto Encyclopedia of Genes and Genomes (KEGG) was utilized to analyze metabolic pathways, gene functions, and gene interactions under conditions of delayed salt stress. KEGG pathway unigenes were identified and annotated for each exposure time (6 h, 12 h, 24 h, 48 h, 72 h) (Table S8, S9, S10, S11, and S12). The top thirty KEGG enrichment DEGs were recorded at each delayed stress time during the study (Fig. 6). The most prominent KEGG enrichment pathways included plant hormone signal transduction, *Mitogen-Activated Protein Kinase Kinase Kinase (MAPK)* signaling pathway, photosynthesis-antenna pathway, ribosomes, glycolysis/gluconeogenesis, antigen processing, starch and sucrose metabolism, circadian rhythm-fly, protein export, and DNA replication were identified (Fig. 6). The total number of KEGG annotation unigenes varied for each exposure time, ranging from 1100 (8% ratio) at 12 h to 3986 (30.11%) at 72 h. Additionally, 283 to 384 reference pathways were identified at each exposure time. The variation in the number of pathways may be attributed to the activation of different adaptive mechanisms under salt stress conditions at different exposure times. The major KEGG pathways belonged to "metabolism," "genetic information processing," "environmental information processing," "cellular process," "organismal systems," and "human diseases." The most prevalent pathways at each delayed salt stress time included plant signal transduction hormone and MAPK signal transduction pathways presented in (Fig. S2).

### Identification and classification of salinity inducible DEGs

The DEGs in at vegetative stage was identified based on criteria log2fod > 1 for up-regulation and for down-regulation log2fold change < − 1 and FDR < 0. 001.

### Regulatory genes

The study aimed to investigate the activation patterns of regulatory protein kinases and plant hormones in response to salt stress during the vegetative stage in faba bean. The investigation revealed a diverse array of regulatory protein kinases that were elicited at distinct time points during salt stress exposure. Notably, the *Mitogen-activated Protein Kinase (MAPK)* class exhibited the highest abundance across all exposure durations. Furthermore, the study discerned differential activation profiles of various plant hormones under different exposure time salt stress duration. Among these abscisic acid (ABA) emerged as the most prominently activated hormone. A subset of these DEGs was closely associated with plant hormones such as *gibberellic acid* (GA), *Jasmonic acid* (JA), *cytokinin* (CK), *salicylic acid* (SA), and *auxin* (IAA). Notably, an in-depth examination of auxin-related DEGs indicated that the tryptophan aminotransferase-related protein class predominated among these genes, with peak exposure time point. Intriguingly, the number of DEGs declined as the duration of salt stress increased, indicative of adynamic regulatory response (Table S13).

### Functional proteins kinases

**Figure 7:**
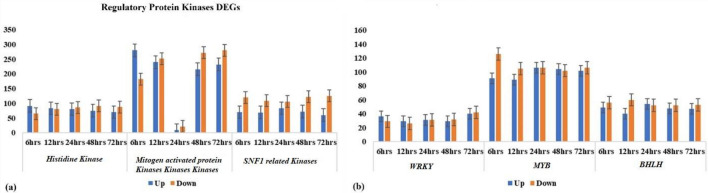
(**a**) Distribution of differentially expressed protein kinases (*histidine Kinase*, *mitogen-activated Kinases Kinases*, and *SNF1-related Kinases*) and (**b**) transcriptional factors (*WRKY, MYB,* and *BHLH*) at different time points (6 h, 12 h, 24 h, 48 h, 72 h) of 200 mM salt stress in Faba bean genotype Hassawi-2.

Likewise, DEGs implicated in “detoxification” encompassed genes associated with respiratory burst, peroxidase, and glutathione S-transferase activities. Among these, the highest number of upregulated DEGs belonged to the “peroxidases” category, with a range of 16 to 29 genes, while downregulated DEGs numbered between 12 and 44 demonstrating variability in response across different time points of delayed stress exposure in faba bean during the vegetative stage (Table S14). The profound influence of oxidative stress on plant physiology underscores the evolutionary adaptation of plants in developing enzymatic defenses to safeguard themselves against oxidative damage. Plants exhibit the remarkable capacity to discern stress cues and transmit specialized stress signals, thus eliciting a precise and coordinated cellular and molecular response. This orchestrated response culminates in the activation of DEGs that are intricately involved in critical processes including osmotic adjustment, detoxification, osmotic protection, and LEA protein collectively enhancing their ability to withstand and mitigate the deleterious effects of oxidative stresses. These DEGs include *arginine decarboxylase*, *Choline monooxygenase*, *Pyrroline-5-carboxylate synthase*, *S-adenosylmethionine decarboxylase*, *Trehalose-6-phosphate synthase*, *Aldehyde dehydrogenase family 7-member*, *Trehalose-6-phosphate synthase*, *Trehalose-phosphate phosphatase*, and *Galactinol synthase* (Table S14). The various classes of functional proteins, such as *histidine kinase*, *mitogen-activated protein kinase kinase kinase*, and *sucrose non-fermenting 1 (SNF1),* were found to be differentially expressed at different exposure times to salt stress (Fig. 7a). Both up and down-regulation of these proteins were observed.

**Figure 8:**
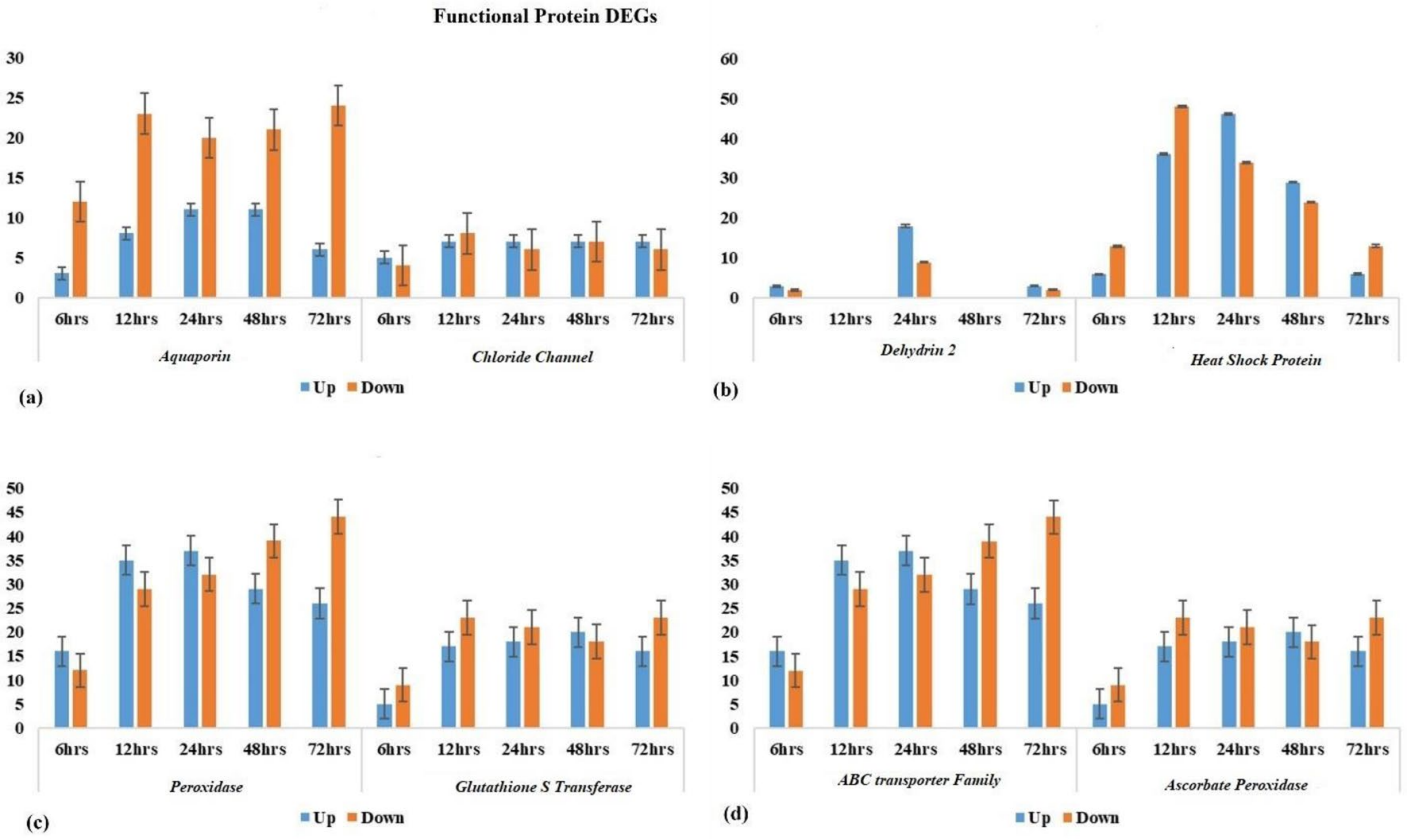
Distribution of differentially expressed functional proteins (**a**) aquaporin and chloride channel, (**b**) dehydrin-2 and heat shock proteins, (**c**) peroxidase and Glutathione S transferase, (**d**) ABC transporter protein family and Ascorbate peroxidase at different time points of salt stress.

Arginine decarboxylase DEGs exhibited up-regulation at 6 h, 12 h, 24 h, 48 h, and 72 h of salt stress exposure compared to the control. Similarly, the differentially expressed genes (DEGs) encoding for *ascorbate peroxidase* were initially up regulated at 6 h of salt stress. However, with increasing exposure to salt stress, the number of DEGs decreased, indicating down-regulation of ascorbate peroxidase (Fig. 8d). The most abundant functional protein classes for osmotic adjustment were *S-adenosylmethionine decarboxylase*, *Trehalose-6-phosphate synthase*, and *Trehalose-phosphate phosphatase*, which were found at each salt stress time. Similarly, the most abundant class of DEGs (up and downregulated) involved in detoxification were *peroxidase*, followed by *respiratory burst oxidase*, and *glutathione S-transferase* (Fig. 8c). For osmotic protection, significant DEGs (up and downregulated) related to aquaporins were also found, followed by *ABC transporter* family proteins (Fig. 8d) and *chloride channels*, respectively, at all salt stress exposure times (Table S14).

The contemporary transcriptome profiling of faba bean under salt stress conditions has unveiled a repertoire of distinct transcription factors (TFs), encompassing *WRKY, BHLH, AP2-EREBP* and *MYB* TFs. These TFs play pivotal roles in orchestrating responses to both biotic and abiotic stress, mediating cell signaling cascades, governing developmental processes, and modulating secondary metabolic pathways. Of note, the *BHLH* TF emerges as a multifaceted regulator involved in diverse biological context, including skeletal muscle development, embryogenesis, neurogenesis, stem cell maintenance, and early-stage developmental processes. Within this context, the study has identified both upregulated and downregulated DEGs for *WRKY*, *MYB*, and *BHLH* DEGs were found (Fig. 7b). Remarkably, among the TFs, MYB emerged as the most abundantly expressed TF protein across all time points (6 h, 12 h, 24 h, 48 h, and 72 h) during exposure to salt stress, followed by *BHLH, WRKY*, and *AP2-EREBP* (Fig. 7b). LEA proteins were also found, with the most abundant class being HSPs [(6–13), (36–48), (46–34), (29–24), and (6–13)], which were upregulated and downregulated at 6 h, 12 h, 24 h, 48 h, and 72 h, respectively. The maximum number of DEGs of LEA proteins was found at 24 h of salt stress exposure, including *dehydrin-2*, dehydration-related proteins, and HSPs (Fig. 8b).

#### Quantitative RT-PCR (qPCR) analysis

Ten potential target DEGs genes were selected from the RNA-seq data for qRT-PCR validation to confirm the expression of target genes based on their potential functions. The selected genes were *calcium-dependent lipid-binding* (c116987_g1_i1-3), *phospholipase* (c141730_g3_i3), *CBL-interacting serine/ threonine-protein kinase* (c126419_g1_i2), *respiratory burst oxidase* (c128033_g2_i1-11), *catalase* (c123109_g1_i1), *ABC transporter family protein* (c144845_g1_i1), *Chloride channel* (c96128_g1_i2), *heat shock protein* (c131372_g1_i1), *BHLH* (c116343_g1_i1), and *Ethylene response transcription factor* (c142959_g1_i1) (Table S15). The qPCR results are consistent with RNA-Sequencing results demonstrating the validity of the study's findings (Table S15).

## Discussion

The Kingdom of Saudi Arabia (KSA) faces hot Mediterranean climate and prevailing abiotic stress conditions. Salinity has a significant impact on yield and productivity in faba beans in KSA. Hassawi-2 (H2) is a salt tolerant genotype most widely cultivated in central and western Saudi Arabia^26^. The current study aims to get deep transcriptome profiling and characterization of DEGs genes under salt stress for different time exposure at early vegetative stage using RNA-Sequencing. The Illumina Hiseq platform was used for RNA-Seq and transcriptome analysis to accomplish the aims. Recently faba bean genome has been discovered fully. Many studies have used RNA-Seq technique to develop transcriptome profiles effectively, frequently using closely related species as a reference genome^27^. Previous studies on faba bean transcriptome were performed only for, gene function expression profile of Faba bean (Vicia faba) Seeds^28^ salinity tolerance during seed germination^6^, drought stress^29^, vernalization^30^ and cold stress responsive genes^31^. The current study, therefore, is the first report on faba bean leaves at early vegetative stage under salinity stress conditions using de novo assembly.

There were total assembled raw reads of 111,425,427 bp while clean reads of 94,683,004 bp were found in the Faba bean leaves at five times salt stress exposure. Similar results were reported by using the RNA-Seq and transcriptome analysis investigation in faba bean leaves under drought stress conditions and recorded 657.2 M reads with 606.35 clean reads^27^. Similarly, the current study recorded 174,051 transcripts with an average GC (38.31%), N50 (941), and average contig length 640.19, while recorded 140,308 unigenes, with an average GC (37.78%), N50 (1,038) and average contig length (674.82). Previous studies on faba bean recorded, 47,621 unigenes across all faba bean genotype with an averge mean 38,712 unigene per genotype^26^, 41,049 transcripts with unigene 18052 from pooled samples (2 faba bean genotypes)^32^, in Fiord (faba bean genotype) 21,297 contigs with 17,160 average unigene in transfer cell development stage^23^, biotic stress at leaves stage in faba bean recorded 393 and 457 over-expressed transcript was found in response to infection^22^. The faba bean transcriptome assembly in leaves recorded substantial GC content in the current study for 6 h (46%), 12 h (45%), 24 h (45%), 48 h (45%) and 72 h (43%) respectively under sainilty stress. The similar results were recorded in different other crop plants transcriptome profiling studies of potato (39–46%), Pea (42–45%), and arabidopsis (43–46.6%). While higher GC content was recorded in tobacco (41.8–47.4%), tomato (41.7–47.2%), rice (54.2–67%), maize (55.8–67.4%) and barley (55.2–66%)^33^. The all unigenes set were aligned by using the different functional databases. Of the total unigenes the most significant similarity was observed top eight species distribution for functional annotations are *Arabidopsis thaliana* (67.24%), *Nicotiana tabacum* (3.15%) and *Oryza sativa subsp. Japonica* (3.13%), *Homo sapiens* (2.10%), *Drosophila melanogaster* (1.85%), *Pisum sativum* (1.57%), *Mus musculus* (1.40%), *Glycine max* (1.24%), and others include (16%) in the database. The contrasting results were recorded during Faba bean transcriptome analysis under drought stress conditions with species annotation of 46.85% belong to *medicago truncatula,* and 26.99% to chickpea^29^.

The current study found unmapped reads at each salt stress level viz; 18.50%, 19.44%, 19.55%, 20.39%, 21.52; and 22%, respectively across all runs. However, Yang et al.^26^ found the proportion of unmapped reads consistently low (2.5%) in Faba bean under salt stress at germination stage and found the correlation between the RPKM values of individual libraries within the of 0.92–0.98 range. These characteristics enabled us to analyze detail depth of the RNA-Seq data under salt stress condition at early vegetative development stage.

Based on pairwise comparisons between control and salt-stressed samples, the number of DEGs was found to be substantial and varied with the length of salt treatment. Elevated DEGs at nearly all time points indicate critical gene expression alterations in response to salt stress. The greatest amount of DEGs were found high at 72 h of salt exposure (Fig. 3). Upregulated genes increased with increasing time exposure of salt stress at all exposure time points (Fig. 3). Similarly increasing pattern of down-regulated genes were observed except 24 h salt exposure, where down-regulated genes were less than 12 h of salt stress. These findings are in line with other studies on transcriptional responses in leaf tissues of several plant species under abiotic stressors^34,35^. The large number of genes that are downregulated in leaves exposed to high salinity may be a result of the leaves ability to conserve energy and resources by suppressing the transcription of genes primarily linked to oxidative processes and cell wall compartments.

To further elucidate the mechanisms of faba bean salt stress tolerance at vegetative stage, DEGs at all time points salt exposure were functionally characterized into different GO categories and KEGG pathways. The GO functional annotations result biological process, molecular function, and cellular components categories (Fig. 4) recorded a wide range of activities related to plant growth, development, and stress tolerance mechanisms in faba bean (Fig. 5). Similar unigenes were found in lentil transcriptome profiling^36^, Fababean^37^, *Arabidopsis*^38^ and other legumes crop^39^. Likewise, several salt-induced pathways have been activated including MAPK signaling pathway (ethylene response factor, osmotic stress management), calcium signaling pathways, plant hormone signal transduction, phospholipase C signaling and Ras signaling pathways as summarized in Fig. S2. These salt-induced pathways have been previously identified for Cowpea^35^, *Cenostigma pyramidale*^40^, and rape seed^41^.

The present study identified the salt-inducible genes previously associated with salt stress. These salt inducible genes can be broadly classified into two groups. The first group is comprised of regulatory proteins, i.e., these include, protein kinases, protein phosphatases, enzymes involved in phospholipid metabolism, other enzymes involved in hormone such ABA and ethylene response and various transcription factors. The second class includes proteins that most probably function in abiotic stress tolerance. These include molecules such as key enzymes for osmolyte biosynthesis, sugar and proline transporter, water channel proteins, detoxification enzymes, chaperones, LEA proteins, and other proteins (Table S13). In regulatory proteins, majority DEGs encoded kinases proteins family at different delayed stress time exposure. The most abundant class *histidine kinase-5*, *calcium ion binding*, *mitogen activated protein kinase kinase kinase*, *sucrose nonfermenting 1 (SNF1) related kinase leucine-rich repeats receptors receptor like kinase (LRR-RLK)*, *Phospholipase A1-Igamma3*, *CBL-interacting serine/threonine-protein kinase*, and *CBL-interacting kinase* were found to be differentially expressed (Table S14). *LRR -RLK* known for abiotic stress response specifically salinity stress. It was also suggested that LRR reduced the salt sensitivity and enhanced the salt tolerance^42^. *Sucrose nonfermenting-1 (SNF1)-related protein kinases (SnRKs)* also an important class of protein kinases showed significant roles for metabolism management under abiotic condition, providing a channel for metabolic and stress signaling to interact, occasionally *abscisic acid (ABA)* involved as stress hormone^43^. Similarly, another important class *LRR-RLK* were also found in all delayed stress faba bean samples. *LRR-RLK* is considered a large complex gene group in crop plants and has their role for the development in stress response. *LRR* receptors are composed of transmembrane, intracellular kinase and extracellular domain that help to regulate stress management^44^. *RLKs*, have significant roles in regulating plant developmental processes, signaling networks, and disease resistance. According to previous studies, numerous *RLKs* have been found to participate in responding to abiotic stress, such as *ABA* response, *Ca*^*2*+^ signaling, and antioxidant defense^45^. Likewise, protein kinases interact with *CBL* are significant for stress adaptation. *CBL*-interacting protein kinases confirm their role to increase resistance under osmotic and salinity stress condition^46^.

Similarly, different DEGs which regulate the plant hormones such as *ABA*, and *aldehyde dehydrogenase*), *ethylene*, *GA*, *JA* (*linoleate 13S-lipoxygenase*), auxin (*tryptophan aminotransferase related protein*) and auxin response factor, were also differentially expressed under salinity stress condition. Phytohormones i.e., *ethylene* and *ABA* known as stress hormones, regulates plant growth by stimulating their key role in response to biotic and abiotic stress^47^. The similar DEGs related to regulatory and functional proteins were reported in other legumes such as chickpea^48^, Faba bean^6^, *Sinorhizobium meliloti*^49^. Though regulatory elements in these biological processes result in complicated and overlapping responses, *ABA* and *ET* both have their own biosynthesis and signaling pathways^50^.

A large of DEGs encoding transcription factors (*bHLH, NAC, MYB, WRKY*) were also identified at all time points of salt stress (Fig. 7b). Transcriptions factors are broadly categorized as early-induced genes, which are targeted by *proteins kinases* (*PKs*) and phosphatases^51^. Amongst the genes of the TFs, the *bHLH* genes were largely exhibited down-regulation under conditions of salt stress. However, some *bHLH* like *bHLH122* was found to be induced as previously reported role in salt tolerance in *A. thaliana*, where it increases levels of ABA by repressing the catabolism of *ABA*^52^. NAC factors are also played diverse roles in stress responses^53^.

The second class includes proteins that ultimately respond in abiotic stress tolerance. These include molecules such as key enzymes for osmolyte biosynthesis, sugar and proline transporter, water channel proteins, detoxification enzymes, chaperones, *LEA* proteins, and other proteins. Among functional proteins, LEA are group of gene activated under salinity condition that regulate the stress mechanism^54^. Among the osmotic adjustment *trehalose-6-phosphate synthase*, *trehalose-phosphate phosphatase* and *pyrroline-5-carboxylate synthase* were the most abundant DEGs. Eight genes were downregulated at 6 h, 24 h while 7 and 9 genes were downregulated at 12 h, 48 h and 72 h delayed time.

Aquaporins are the main participants in the mechanism through which cells can maintain their homeostasis under abiotic stress i.e., salt conditions by enhancing the passage of water through membranes. Throughout all stress times, transcripts associated with water channels “*aquaporins*”, were detected in the current study. The current study showed both up and down regulated DEGs for aquaporins and showed their transcriptional behavior. Chloride channels play a role in regulating transepithelial transport, controlling membrane excitability, and maintaining cell volume as well as pH levels within both intracellular and intra-organelle environments^55^. As the duration of salt stress exposure increased, the number of up-regulated DEGs associated with aquaporin and chloride channels also largely increased, indicating changes in transcript behavior (Fig. 8a). The similar transcript change behavior were found in Prunus^56^, Canola^57^, and Soybean^58^ under salt stress condition. Similar osmotic adjustment classification under salinity stress were found in woody legume^59^, alfalfa^60^ and chickpea^61^ under salinity stress. Some genes were present related to transcript, translation, ribosomal structure, and biogenesis. These genes functions probably resulted in varied salt stress responses and regulate the mechanism under abiotic stress condition^62^.

This study also found antioxidants enzymes i.e., *ascorbate peroxidase*, *glutathione S-transferase*, *glutathione peroxidase* and *superoxide dismutase*, largely differentially expressed at each delayed time under salt stress condition (Table S13). The role of detoxification enzyme to enhance the cell protection process under salt stress well reported in soybean^63^, chickpea^64^, and Kenaf^65^. Therefore, differential expression of these DEGs which regulate the functional proteins suggests an important role in salt stress response. In addition, a substantial proportion of the DEGs identified in this study are uncharacterized. However, many of these DEGs exhibited a considerable change in expression levels throughout the delayed stress time (Table S13). Therefore, these DEGs are very useful resource that might regulate specific responses to salt stress and others stress in faba bean. Further analysis of these transcripts will be helpful for our understanding of salt tolerance mechanisms in plants.

## Conclusion

This study underscores the paramount importance of addressing abiotic stresses in global plant production. The study has made significant strides in unraveling the molecular intricacies underlying salt tolerance. This study is the first report of leaf transcriptome profiling in an economically important legume crop faba bean (*Vicia faba* L.) at early vegetative stage under salt stress conditions. Within this research, we have successfully identified several putative key genes and pathways associated to the faba bean's salt tolerance mechanism. These includes DEGs related to osmotic adjustment, ion homeostasis, antioxidant defense, hormonal, and transcriptional regulation. These DEGs in the faba bean genome activates various adaptive mechanisms when confronted with salt stress. These findings significantly enhance our understanding of how faba bean plants genetically respond to salinity stress, laying the foundation for advancements of molecular breeding techniques to improve the productivity of salt-tolerant genotypes under salinity stress.

## Materials and methods

### RNA extraction, and sequencing

The Hassawi-2 salt-tolerant faba bean^26^ genotype was used for transcriptome profiling using RNA-Seq quantification. The Hassawi-2 genotype seeds were grown in sandy soil in the pots. The growth chamber condition (28 °C) and 10/14 h light/dark were maintained. One seedling per pot was maintained for proper growth and development. The seedlings were maintained at three leaves stage with three biological replicates for each delayed time. The 200 mM salt stress was applied at three leaves stage and plantlets were exposed to salt stress for five delay time exposure 6 h, 12 h, 24 h, 48 h and 72 h along with control. The collection of plant samples/parts under control and salt stress condition complies with national, international and/or institutional guidelines in the present study^66^.

The samples were collected from control and 200 mM stress delayed time and stored the sample in RNA later (Invitrogen; Thermo Fisher Scientific, USA) to maintain the quality and integrity of each sample. 18 samples were outsourced to Macrogen (https://dna.macrogen.com/) for RNA-sequencing. The quality of extracted RNA was evaluated on Agilent Bioanalyzer 21,000 and quantity was determined using a NanoDrop Spectro-photometer (Thermo Fisher Scientific, USA). RNA sample with RNA integrity number > 6.5 was further processed for library construction. Finally, the paired-end sequencing on both ends of the cDNA are sequenced on Illumina NovaSeq 6000 platform. The quality of produced data is determined by the Phred quality score at each cycle. The raw reads were trimmed for removal of adapter sequences and low quality sequences using Trimmomatic program (Trimmomatic 0.38) and the retrieved reads after filtering were labeled as "clean reads" and stored in FASTQ^67^ format.

### Denovo transcript assembly

The trimmed reads for all samples were compared and merged into one file to do transcriptome assembly using Trinity program (Trinity version trinityrnaseq_r20140717, bowtie 1.1.2). For assembled genes, the longest contigs are filtered and clustered into the non-redundant transcripts using CD-HIT-EST program (CD-HIT version 4.6) and defined these transcripts as unigenes. Obtained unigenes are used for the subsequent annotation, open reading frame (ORF) prediction using Transdecoder program to identify coding regions and the further differentially expressed genes (DEG) analysis.

### Functional annotation

For functional annotation, unigenes were searched against Kyoto Encyclopedia of Genes and Genomes (KEGG) http://www.genome.jp/kegg/ko.html/ version (v20220103), NCBI Nucleotide (NT) https://www.ncbi.nlm.nih.gov/nucleotide/, NCBI Non-redundant Protein (NR; v20180503) https://www.ncbi.nlm.nih.gov/refseq/about/nonredundantproteins/, Pfam (https://pfam.xfam.org/ (v20160316) Gene ontology (GO) http://geneontology.org/ (v20180319), UniProt https://www.uniprot.org/ (v20180116) and EggNOG http://eggnog6.embl.de/ (eggnog4) using BLASTN of NCBI BLAST https://blast.ncbi.nlm.nih.gov/Blast.cgi?PROGRAM=blastn&PAGE_TYPE=BlastSearch&LINK_LOC=blasthome/ and BLASTX of DIAMOND http://ab.inf.uni-tuebingen.de/software/diamond software with an E-value default cutoff of 1.0E^−5^.

### Differentially expressed genes (DEGs)

The RNA-Seq by expectation–maximization (RSEM) algorithm is employed to estimate the abundance of unigenes across samples. The expression level is determined based on read count. Trimmed reads from each sample were aligned to the were aligned to the assembled reference using Bowtie^68^ program (http://bowtie-bio.sourceforge.net/Bowtie2/index.shtml/). Subsequently, the RSEM algorithm was utilized to calculate the fragments per kilobase of transcript per million mapped reads (FPKM) values for each sample^69^. For the analysis of DEGs, the abundance of unigenes across samples is estimated as a measure of expression using the RSEM algorithm. This expression value is then employed in further DEG analysis. In groups with different conditions, differentially expressed genes are identified through statistical hypothesis testing. The statistical analysis is performed using Fold Change (FC), and exactTest methods provided by the edgeR package for each comparison pair. Significant results are selected based on criteria of |fc|> = 2 and an exactTest raw *p*-value < 0.05.

### DEGs identification and function categorization

Differentially expressed genes (DEGs) were sorted based on read count for unigenes. Furthermore, the data quality and similarity check were done in case of biological replicates. GO annotation and GO functional enrichment analysis was performed using R function phyper. The fold change and hierarchical clustering and exact Test analysis was done using edgeR function. To reduce systematic bias, estimates the size factors from the count data and applies Trimmed Mean of M-values (TMM) normalization with edgeR R library. In different tests and analysis, false discovery rate (FDR) was used to determine the *P* value threshold. Difference in the DEGs threshold value was | log2 ratio |≥ 1, while FDR absolute value ≤ 0.001 was established. For functional annotation of the unigenes, the Gene Ontology (GO) database was applied to classify the annotated unigenes using BLASTX of DIAMOND with an E-value cut-off of 1.0E^−5^. Classification of GO terms were subsequently performed using in-house script. The GO terms belonging to biological process (BP), cellular component (CC) and molecular function (MF) were determined.

### DEGs pathways analysis

The KEGG annotation result was used to classify the DEGs according to their official classification, and the R function Phyper was used to perform KEGG pathway functional enrichment. The FDR for each *p* value was calculated, FDR no longer than 0.001 was recorded as significantly enriched.

### qRT-PCR DEGs validation

Ten significant DEGs were chosen for validation from the RNA_Seq data using qRT-PCR. The primers for these genes were designed with Primer 3 software. The Actin gene served as the internal endogenous control^70^. Total RNA was extracted from Hassawi-2 faba bean genotype, both from control and salinity stress samples. For qRT-PCR reaction of each sample, we utilized the GoTaq 1-Step RT-qPCR system (Promega, USA). The qRT-PCR results were analyzed using the ΔΔCT and 2^−ΔΔCT^ methods to confirm the expression of these genes under salinity stress^71^.

### Supplementary Information

Supplementary Figure 1.

Supplementary Figure 2.

Supplementary Table 1.

Supplementary Table 2.

Supplementary Table 3.

Supplementary Table 4.

Supplementary Table 5.

Supplementary Table 6.

Supplementary Table 7.

Supplementary Table 8.

Supplementary Table 9.

Supplementary Table 10.

Supplementary Table 11.

Supplementary Table 12.

Supplementary Table 13.

Supplementary Table 14.

Supplementary Table 15.

## Data Availability

The RNA sequence data has been submitted to the National Centre for Biotechnology Information (NCBI) (https://submit.ncbi.nlm.nih.gov/subs/sra/) and can be accessed through the Bio Project ID PRJNA943415 and SRA submission ID SUB12947558.
