# Supplementary material for: Transcriptomic analysis reveals candidate genes associated with salinity stress tolerance during the early vegetative stage in fababean genotype, Hassawi-2
Source: Sci Rep. 2023 Dec 1;13:21223. doi: 10.1038/s41598-023-48118-0 (PMC10692206; doi:10.1038/s41598-023-48118-0)
Supplement: Supplementary file 1 — Supplementary Figure 1. [file 41598_2023_48118_MOESM1_ESM.docx]

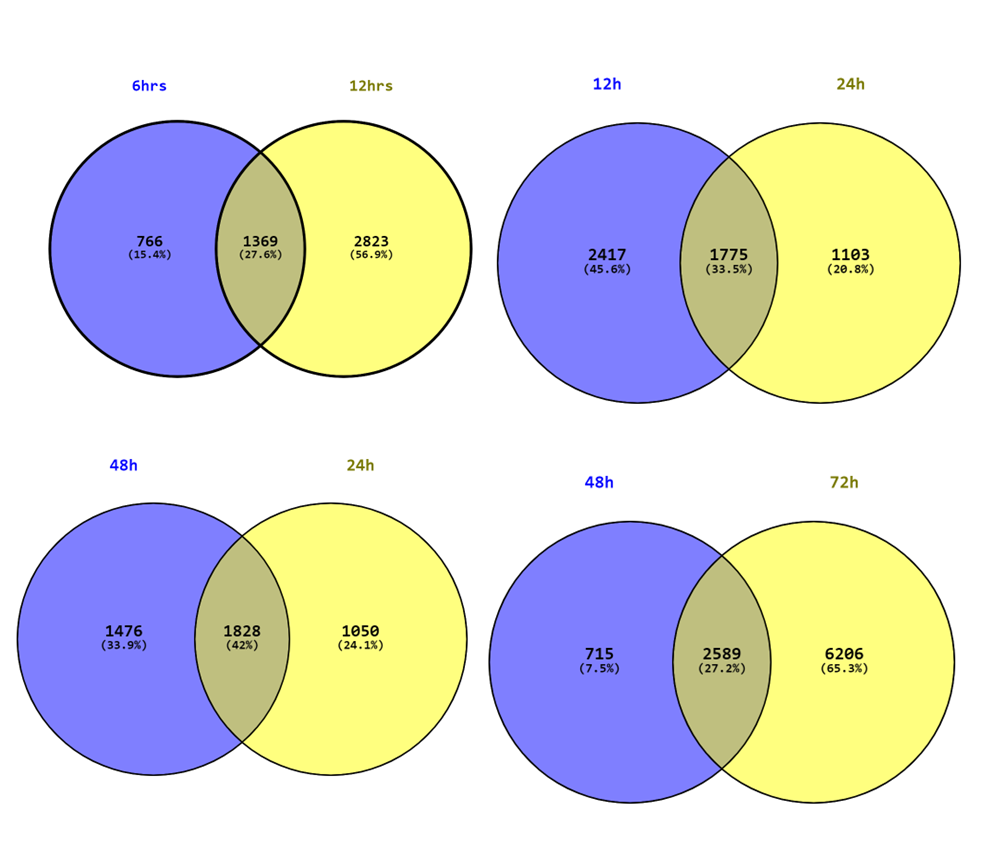


Figure S1. Venn diagram of contrasting combination time exposure under salt stress showing number of DEGs
