# Supplementary material for: Transcriptomic analysis reveals candidate genes associated with salinity stress tolerance during the early vegetative stage in fababean genotype, Hassawi-2
Source: Sci Rep. 2023 Dec 1;13:21223. doi: 10.1038/s41598-023-48118-0 (PMC10692206; doi:10.1038/s41598-023-48118-0)
Supplement: Supplementary file 2 — Supplementary Figure 2. [file 41598_2023_48118_MOESM2_ESM.docx]

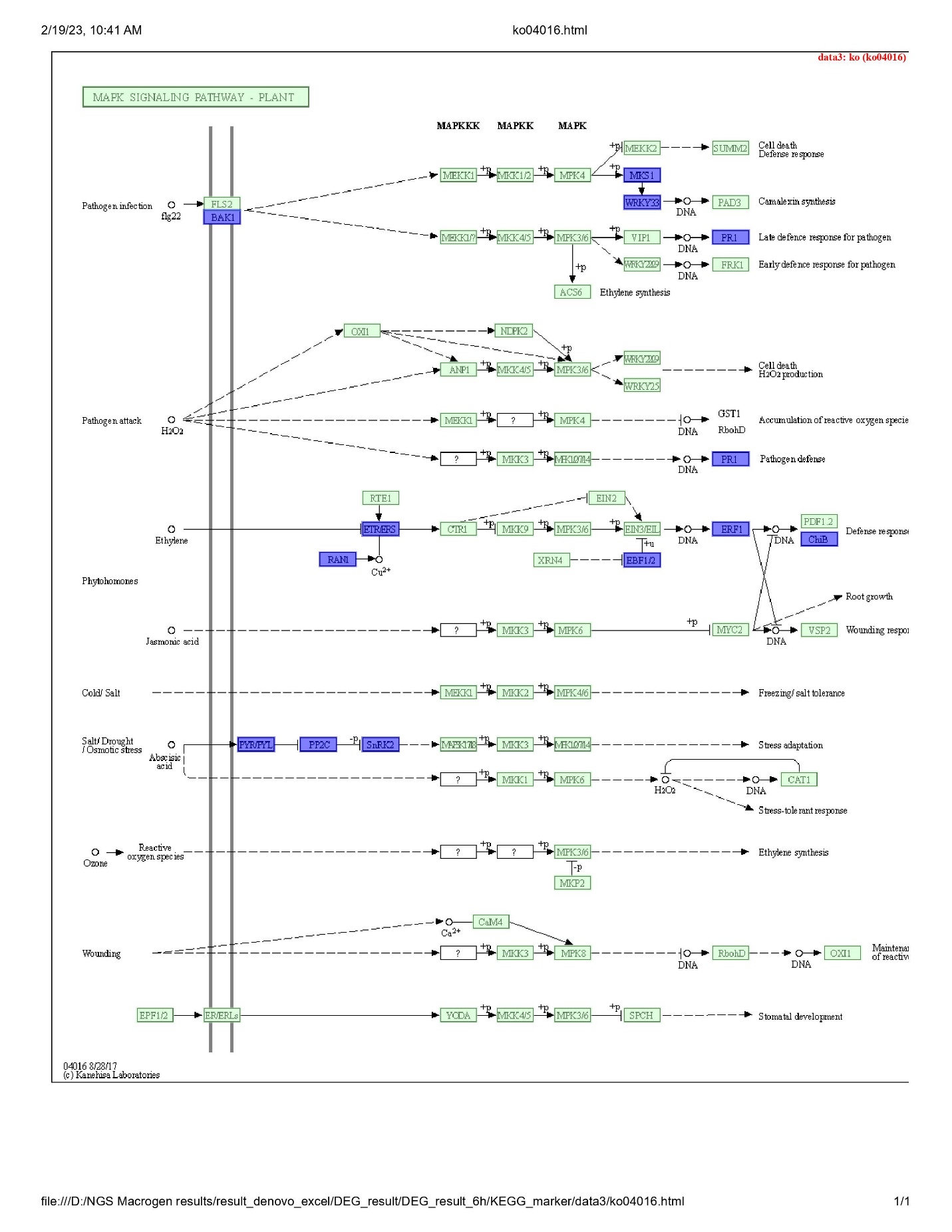


Figure S2.1 MAPK signal pathways response under salt stress at 6 hrs of salt stress. We have secured permission from Kanehisa Laboratories to utilize the KEGG pathway database ^68^


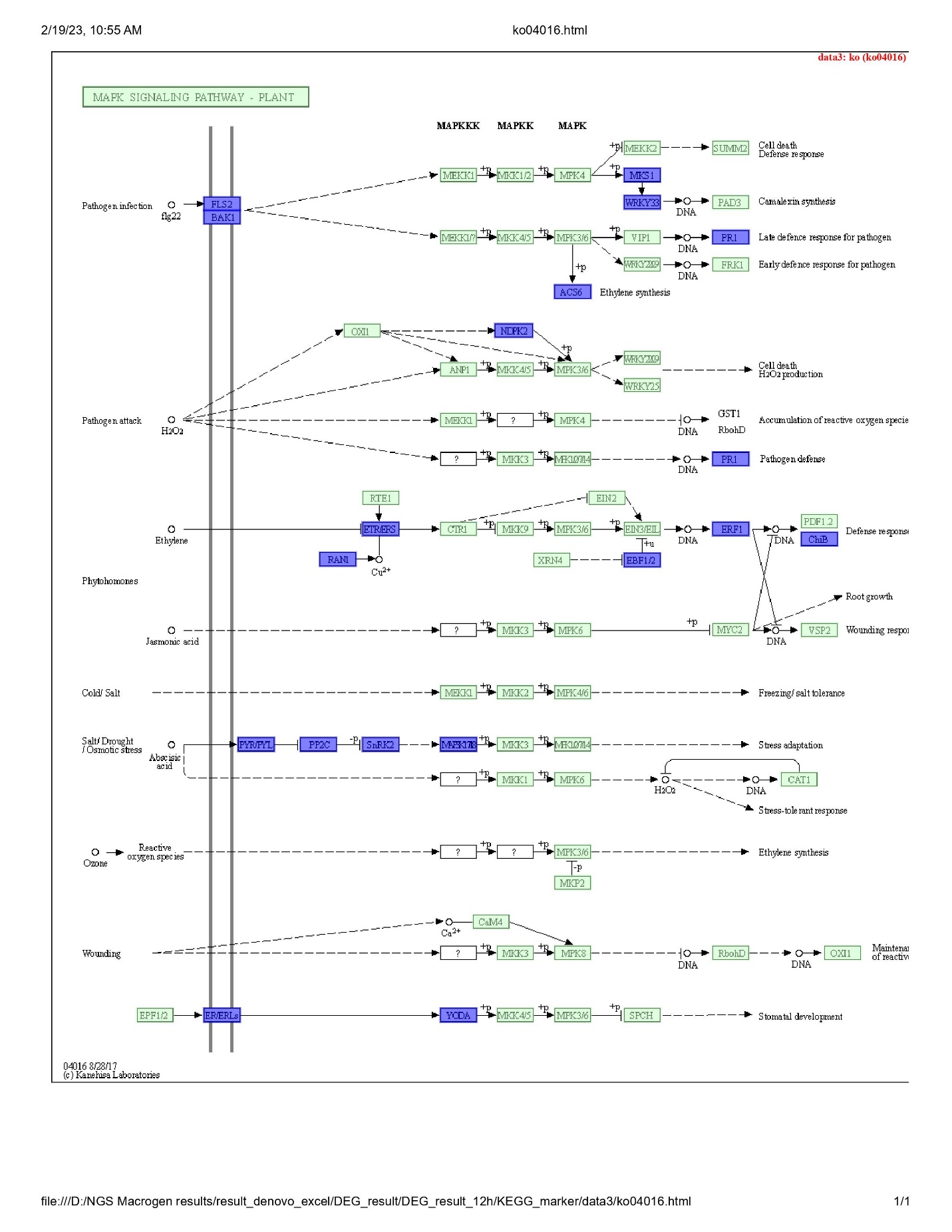


Figure S2.2 MAPK signal pathways response under salt stress at 12 hrs of salt stress. We have secured permission from Kanehisa Laboratories to utilize the KEGG pathway database ^68^


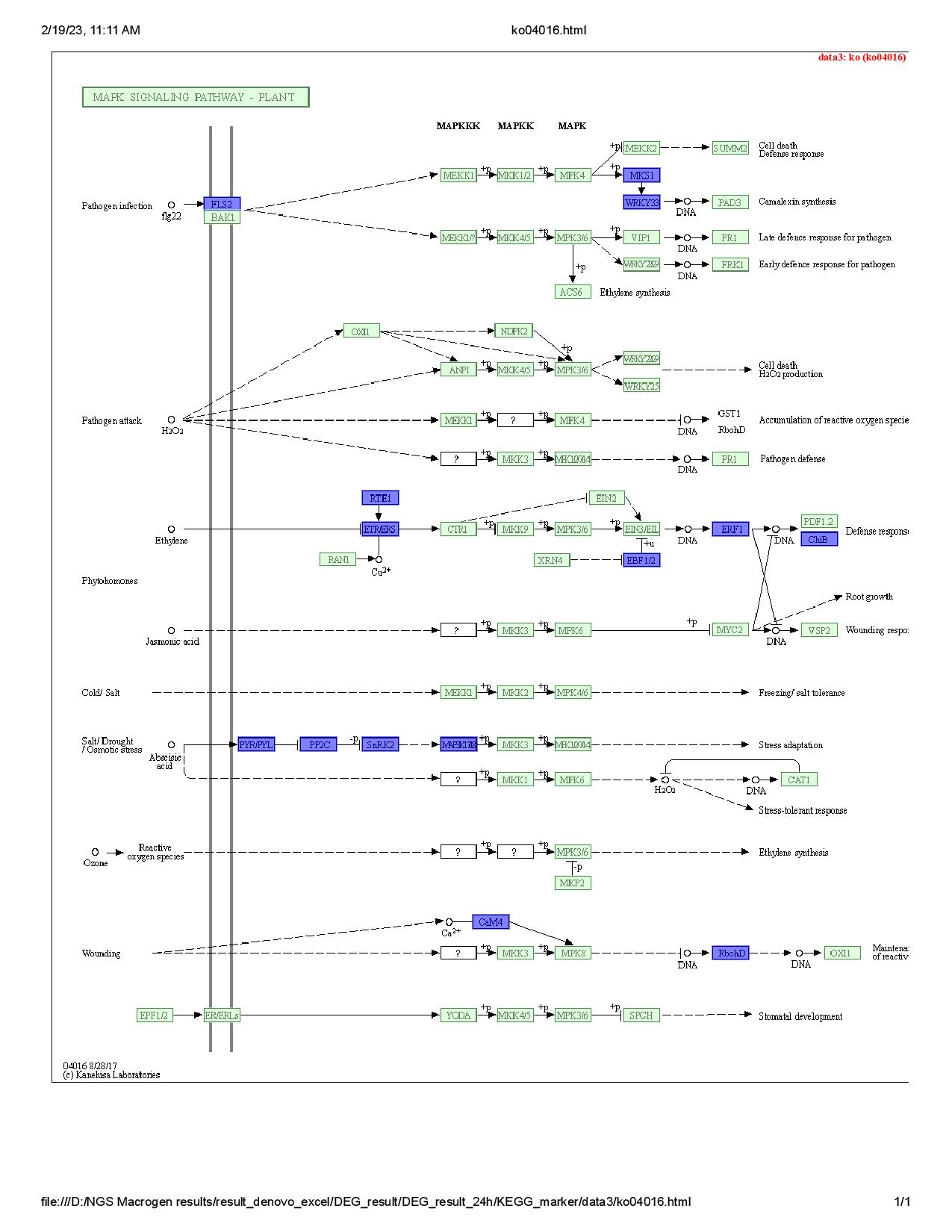


Figure S2.3 MAPK signal pathways response under salt stress at 24 hrs of salt stress. We have secured permission from Kanehisa Laboratories to utilize the KEGG pathway database ^68^


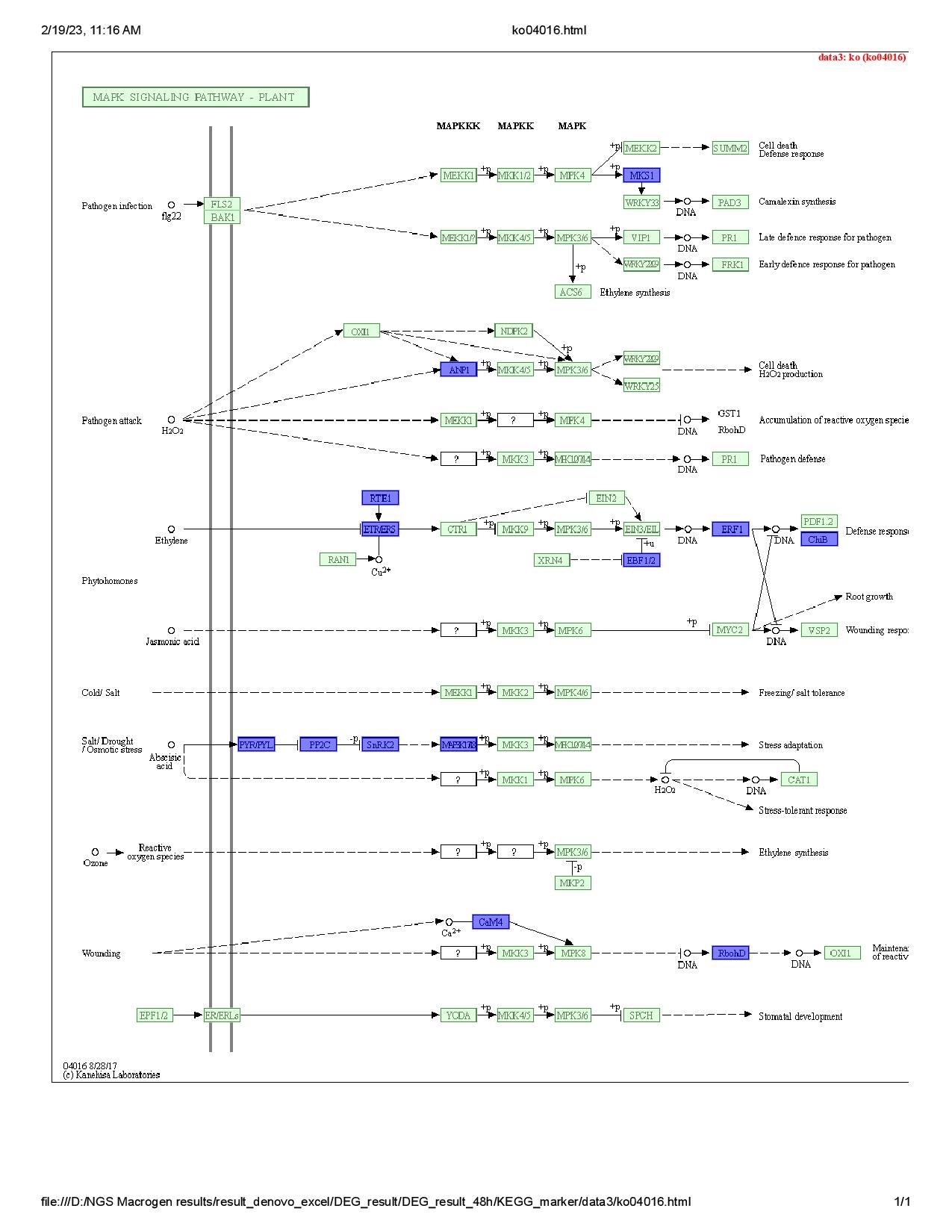


Figure S2.4 MAPK signal pathways response under salt stress at 48 hrs of salt stress. We have secured permission from Kanehisa Laboratories to utilize the KEGG pathway database ^68^


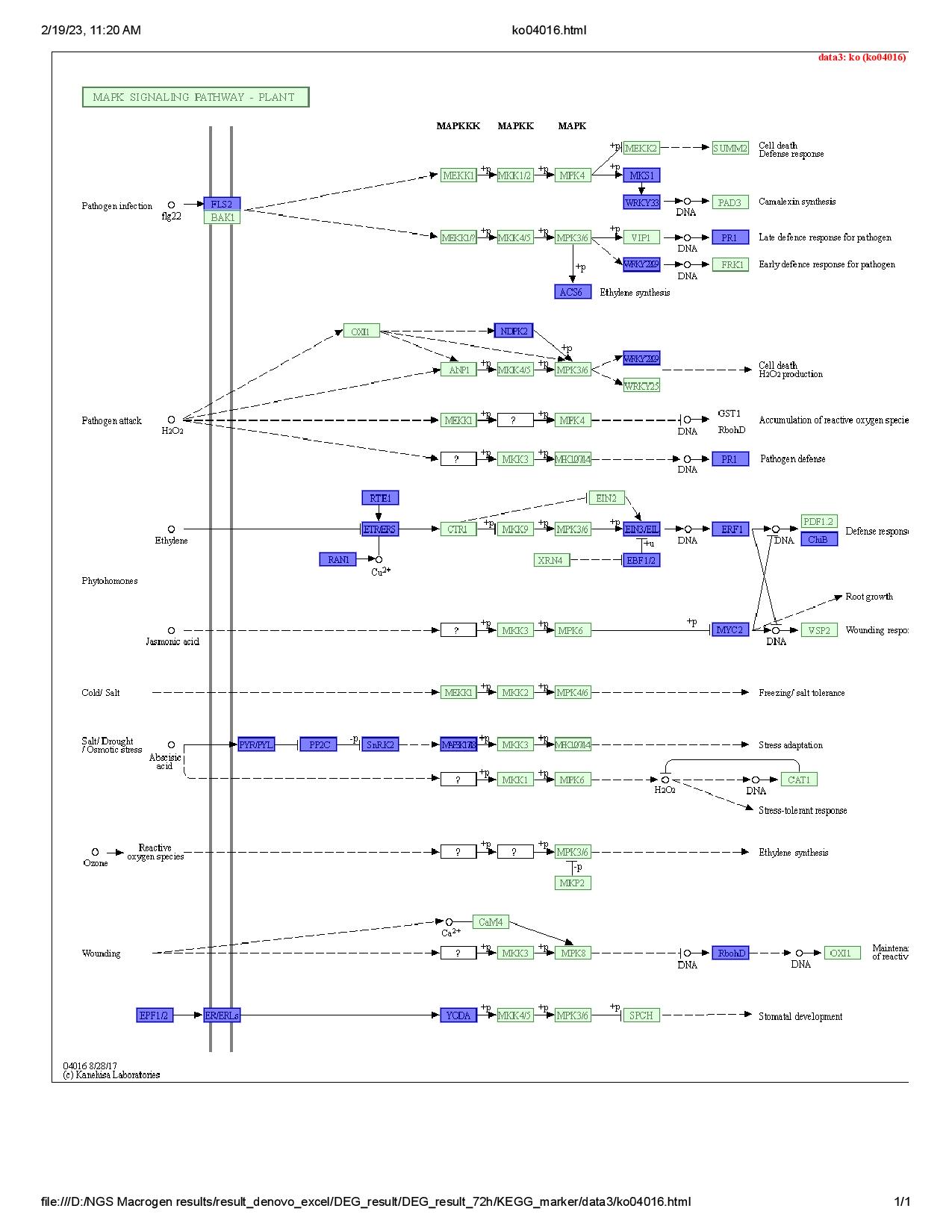


Figure S2.5 MAPK signal pathways response under salt stress at 72 hrs of salt stress. We have secured permission from Kanehisa Laboratories to utilize the KEGG pathway database ^68^


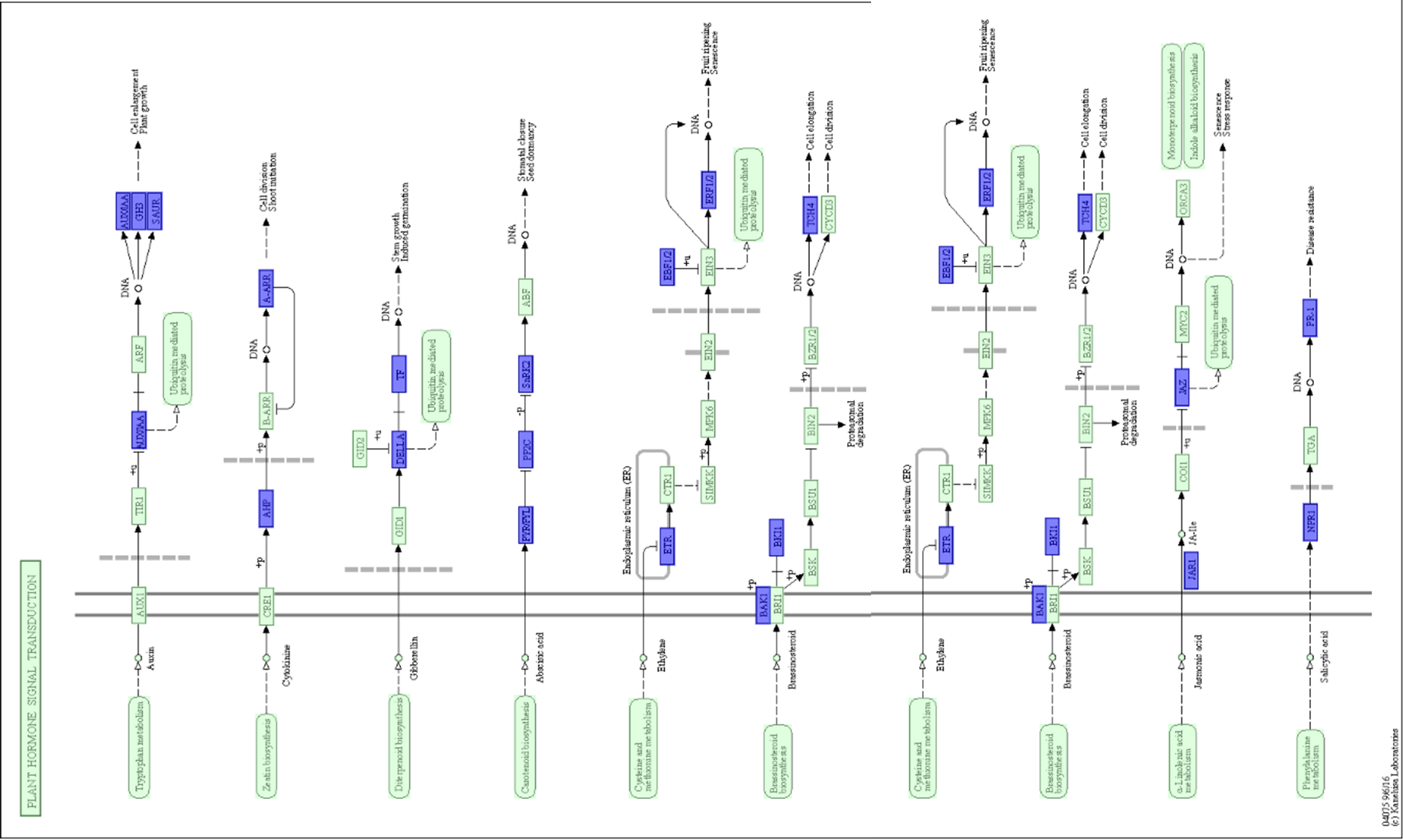


Figure S2.6 Plant hormone signal pathways response under salt stress at 06hrs of salt stress (We have secured permission from Kanehisa Laboratories to utilize the KEGG pathway database)^68^

**
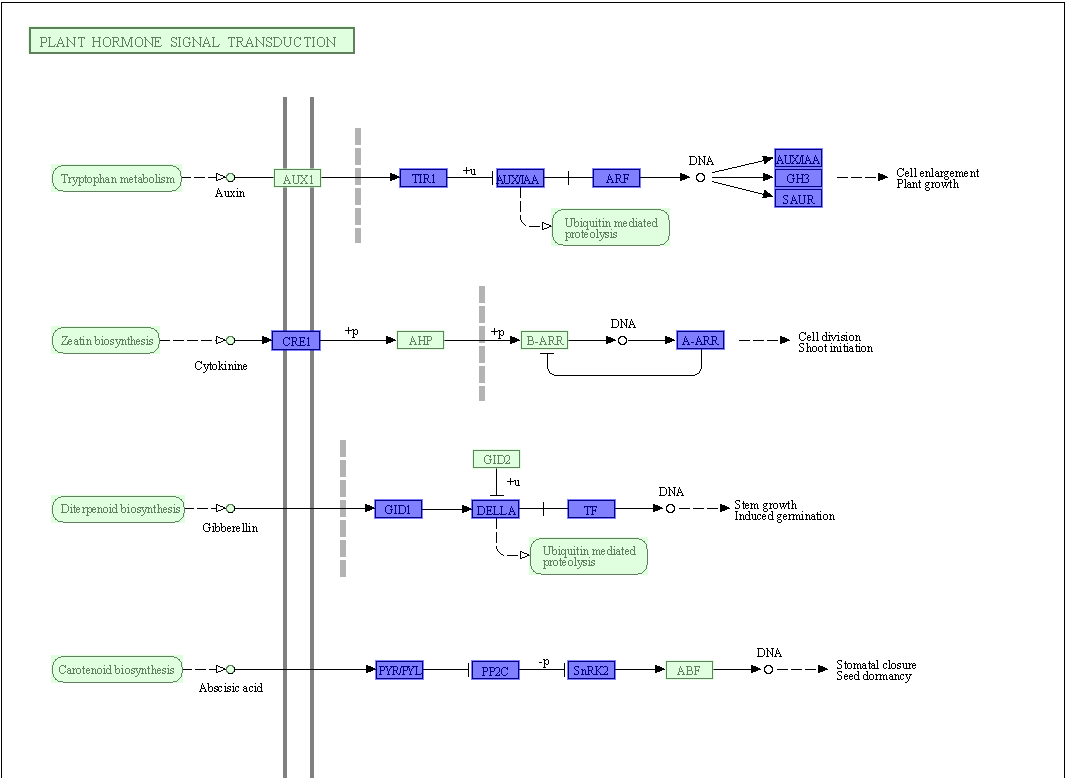
**
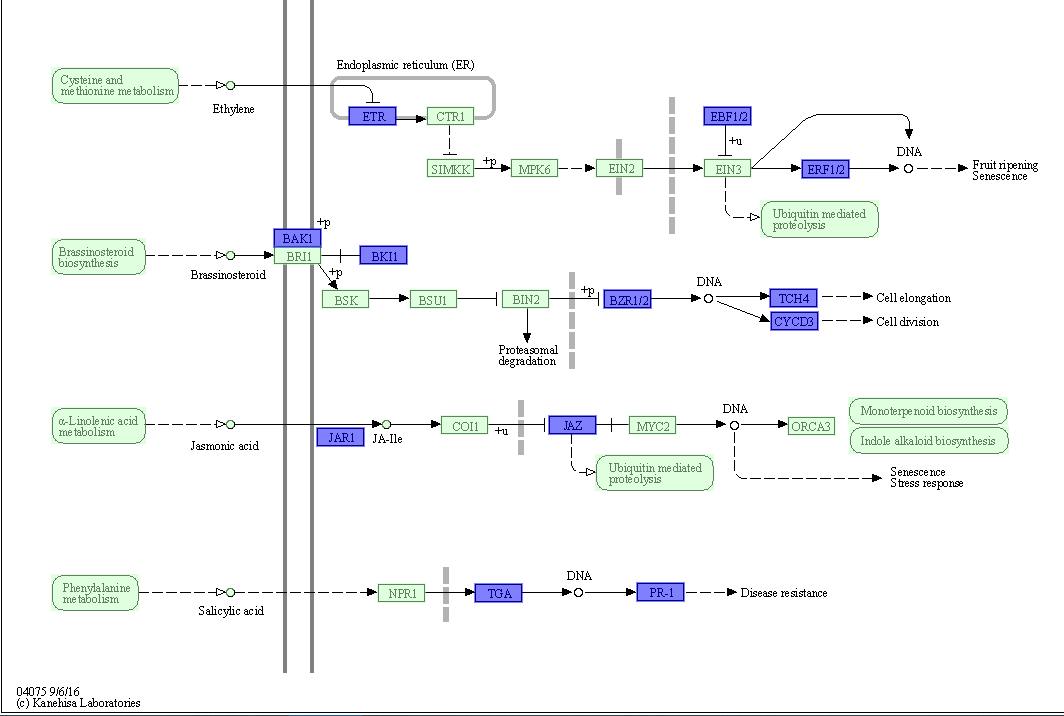


Figure S2.7 Plant hormone signal pathways response under salt stress at 12hrs of salt stress (We have secured permission from Kanehisa Laboratories to utilize the KEGG pathway database) ^68^

**
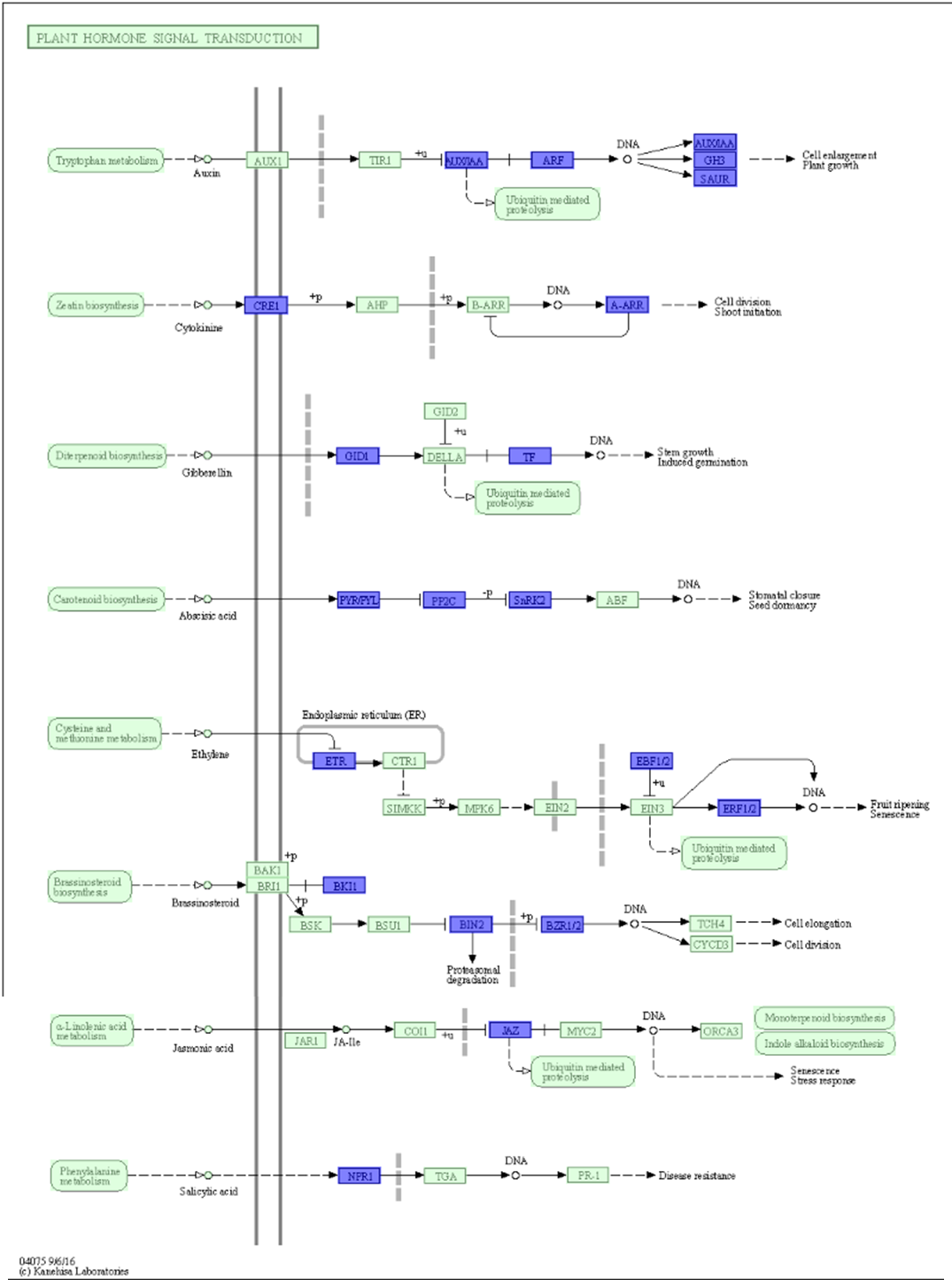
**

Figure S2.8 Plant hormone signal pathways response under salt stress at 24hrs of salt stress. (We have secured permission from Kanehisa Laboratories to utilize the KEGG pathway database) ^68^

**
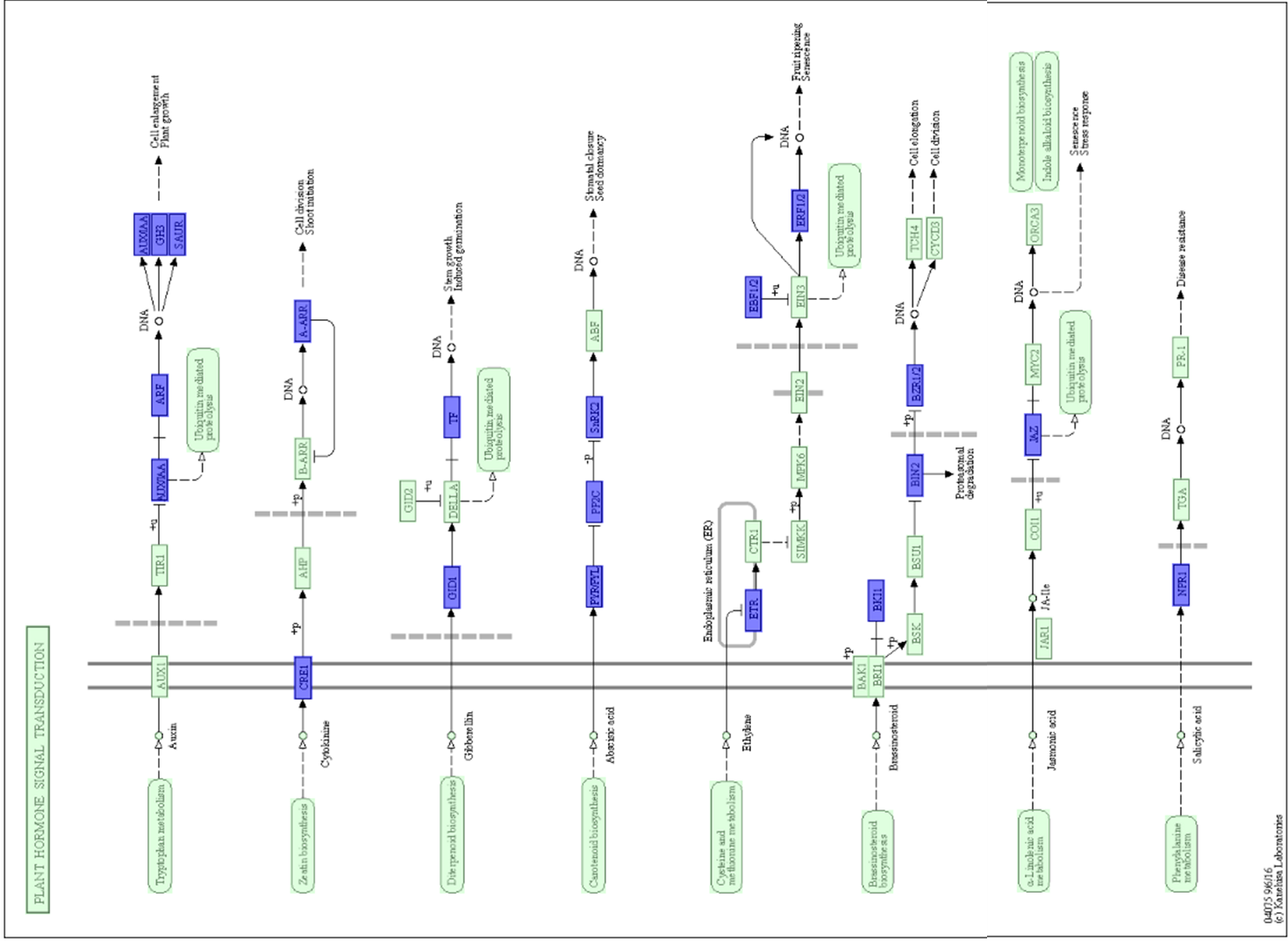
**

Figure S2.9 Plant hormone signal pathways response under salt stress at 48hrs of salt stress (We have secured permission from Kanehisa Laboratories to utilize the KEGG pathway database)^68^.

**
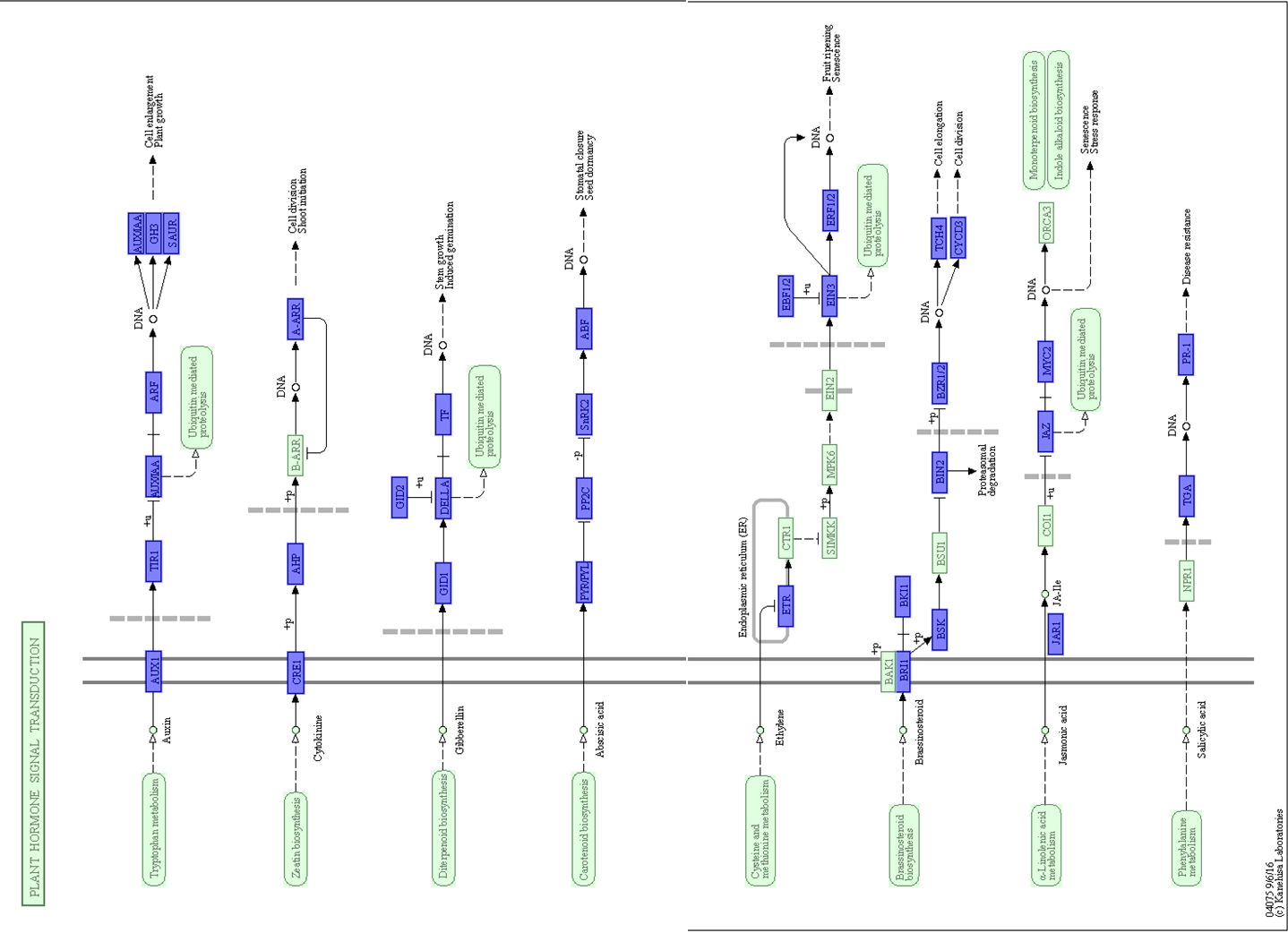
**

Figure S2.10 Plant hormone signal pathways response under salt stress at 72hrs of salt stress. We have secured permission from Kanehisa Laboratories to utilize the KEGG pathway database ^68.^
