# Supplementary material for: Transcriptomic analysis reveals candidate genes associated with salinity stress tolerance during the early vegetative stage in fababean genotype, Hassawi-2
Source: Sci Rep. 2023 Dec 1;13:21223. doi: 10.1038/s41598-023-48118-0 (PMC10692206; doi:10.1038/s41598-023-48118-0)
Supplement: Supplementary file 3 — Supplementary Table 1. [file 41598_2023_48118_MOESM3_ESM.docx]

**Summary of transcriptome sequencing reads in eighteen different samples**

**Table S1.1: Statistics of RNA_Seq data analysis of *Vicia Faba* L. genotype (H2) under different delayed salinity stress**

| File Name | TT  (hrs) | TRRS  (Mbp) | TRS  (Mbp) | GC  (%) | TTRRS  (Mbp) | TTRS  (Mbp) | GC  (%) |
| --- | --- | --- | --- | --- | --- | --- | --- |
| FVC-0h-1_R1.fastq.gz | 0 | 5698.31 | 56.41 | 45.58 | 5585.29 | 55.54 | 45.61 |
| FVC-0h-1_R2.fastq.gz | 0 | 5645.79 | 55.89 | 44.90 | 5541.92 | 55.12 | 44.93 |
| FVC-0h-1_R3.fastq.gz | 0 | 5833.35 | 57.75 | 46.08 | 5706.51 | 56.77 | 46.12 |
| **FVC-0h** |  | 5725.81 | 56.68 | 45.52 | 5611.24 | 55.81 | 45.55 |
| FVS-6h-1_R1.fastq.gz | 6 | 5698.69 | 56.42 | 44.22 | 5574.35 | 55.50 | 44.23 |
| FVS-6h-1_R2.fastq.gz | 6 | 5604.82 | 55.49 | 45.24 | 5466.13 | 54.42 | 45.28 |
| FVS-6h-1_R3.fastq.gz | 6 | 5131.08 | 50.80 | 44.56 | 5033.27 | 50.09 | 44.57 |
| **FVS-06h** |  | 5478.19 | 54.23 | 44.67 | 5357.91 | 53.33 | 44.69 |
| FVS-12h-1_R1.fastq.gz | 12 | 4972.50 | 49.23 | 44.55 | 4867.77 | 48.49 | 44.55 |
| FVS-12h-1_R2.fastq.gz | 12 | 4529.74 | 44.84 | 44.95 | 4448.56 | 44.25 | 44.97 |
| FVS-12h-1_R3.fastq.gz | 12 | 5707.59 | 56.51 | 44.48 | 5609.68 | 55.77 | 44.51 |
| **FVS-12h** |  | 5069.94 | 50.19 | 44.66 | 4975.33 | 49.50 | 44.67 |
| FVS-24h-1_R1.fastq.gz | 24 | 6374.90 | 63.11 | 45.99 | 6232.39 | 61.97 | 46.04 |
| FVS-24h-1_R2.fastq.gz | 24 | 6361.31 | 62.98 | 44.20 | 6208.12 | 61.76 | 44.23 |
| FVS-24h-1_R3.fastq.gz | 24 | 5304.55 | 52.52 | 44.20 | 5227.79 | 51.95 | 44.23 |
| **FVS-24h** |  | 6013.58 | 59.53 | 44.79 | 5889.43 | 58.56 | 44.83 |
| FVS-48h-1_R1.fastq.gz | 48 | 6372.92 | 63.09 | 44.18 | 6216.94 | 61.91 | 44.20 |
| FVS-48h-1_R3.fastq.gz | 48 | 4537.01 | 44.92 | 43.83 | 4469.29 | 44.40 | 43.86 |
| **FVS-48h** |  | 5454.96 | 54.00 | 44.00 | 5343.11 | 53.15 | 44.03 |
| FVS-72h-1_R1.fastq.gz | 72 | 6364.67 | 63.01 | 42.85 | 6253.24 | 62.15 | 42.87 |
| FVS-72h-1_R2.fastq.gz | 72 | 6319.39 | 62.56 | 42.44 | 6202.20 | 61.64 | 42.47 |
| FVS-72h-1_R3.fastq.gz | 72 | 5958.32 | 58.99 | 43.13 | 5864.35 | 58.27 | 43.15 |
| **FVS-72h** |  | 6214.12 | 61.52 | 42.80 | 6106.59 | 60.68 | 42.83 |

FVC= Fababean vegetative control; FVS= Fababean vegetative stress; Name of the RNA_Seq file; TT= Treatment time; TRRS= Total raw reads sequencing; TRS=Total reads sequencing; TTRRS= Total trimmed raw reads sequencing; TTRS= Total Trimmed Reads Sequencing

**Table S1.2. Summary of mapping and unmapped reads data**

| **File Name** | **PR** | **MR** | **% Reads** | **UMR** | **Reads**  **%** |
| --- | --- | --- | --- | --- | --- |
| FVC-0h-1_R1.fastq.gz | 55547958 | 45449884 | (81.82) | 10098074 | (18.18) |
| FVC-0h-1_R2.fastq.gz | 55125126 | 44344678 | (80.44) | 10780448 | (19.56) |
| FVC-0h-1_R3.fastq.gz | 56779694 | 46683476 | (82.22) | 10096218 | (17.78) |
| **FVC0h** | 55817592 | 45492679 | (81.49) | 10324913 | (18.50) |
| FVS-6h-1_R1.fastq.gz | 55508704 | 44600718 | (80.35) | 10907986 | (19.65) |
| FVS-6h-1_R2.fastq.gz | 54425320 | 43556404 | (80.03) | 10868916 | (19.97) |
| FVS-6h-1_R3.fastq.gz | 50096286 | 40719770 | (81.28) | 9376516 | (18.72) |
| **FVS-6h** | 53343436 | 42958964 | (80.55) | 10384472 | (19.44) |
| FVS-12h-1_R1.fastq.gz | 48494372 | 39031498 | (80.49) | 9462874 | (19.51) |
| FVS-12h-1_R2.fastq.gz | 44255750 | 35635894 | (80.52) | 8619856 | (19.48) |
| FVS-12h-1_R3.fastq.gz | 55771808 | 44800574 | (80.33) | 10971234 | (19.67) |
| **FVS-12h** | 49507310 | 39822655 | 80.44 | 9684655 | 19.55 |
| FVS-24h-1_R1.fastq.gz | 61976074 | 50422364 | (81.36) | 11553710 | (18.64) |
| FVS-24h-1_R2.fastq.gz | 61769564 | 48394492 | (78.35) | 13375072 | (21.65) |
| FVS-24h-1_R3.fastq.gz | 51950714 | 41104968 | (79.12) | 10845746 | (20.88) |
| **FVS-24h** | 58565451 | 46640608 | (79.61) | 11924843 | (20.39) |
| FVS-48h-1_R1.fastq.gz | 61911720 | 48575964 | (78.46) | 13335756 | (21.54) |
| FVS-48h-1_R3.fastq.gz | 44407066 | 34857420 | (78.50) | 9549646 | (21.5) |
| **FVS-48h** | 53159393 | 41716692 | (78.48) | 11442701 | (21.52) |
| FVS-72h-1_R1.fastq.gz | 62154594 | 48562292 | (78.13) | 13592302 | (21.87) |
| FVS-72h-1_R2.fastq.gz | 61643858 | 46990012 | (76.23) | 14653846 | (23.77) |
| FVS-72h-1_R3.fastq.gz | 58278666 | 46402328 | (79.62) | 1876338 | (20.38) |
| **FVS-72h** | 60692373 | 47318211 | (77.99) | 10040829 | (22) |

FVC= Fababean vegetative control; FVS= Fababean vegetative stress; PR=Processed Reads; MR= Mapped Reads; UMR= Unmapped Reads
